# Population and single-cell genomics reveal the *Aire* dependency, relief from Polycomb silencing, and distribution of self-antigen expression in thymic epithelia

**DOI:** 10.1101/gr.171645.113

**Published:** 2014-12

**Authors:** Stephen N. Sansom, Noriko Shikama-Dorn, Saule Zhanybekova, Gretel Nusspaumer, Iain C. Macaulay, Mary E. Deadman, Andreas Heger, Chris P. Ponting, Georg A. Holländer

**Affiliations:** 1 MRC Computational Genomics Analysis and Training Programme, MRC Functional Genomics Unit, Department of Physiology, Anatomy and Genetics, University of Oxford, Oxford, OX1 3PT, United Kingdom;; 2 Paediatric Immunology, Department of Biomedicine, University of Basel, and The Basel University Children’s Hospital, Basel, 4058, Switzerland;; 3 Wellcome Trust Sanger Institute–EBI Single Cell Genomics Centre, Wellcome Trust Sanger Institute, Hinxton, Cambridge, CB10 1SA, United Kingdom;; 4 Developmental Immunology, Department of Paediatrics, and the Weatherall Institute of Molecular Medicine, University of Oxford, Oxford OX3 9DS, United Kingdom

## Abstract

Promiscuous gene expression (PGE) by thymic epithelial cells (TEC) is essential for generating a diverse T cell antigen receptor repertoire tolerant to self-antigens, and thus for avoiding autoimmunity. Nevertheless, the extent and nature of this unusual expression program within TEC populations and single cells are unknown. Using deep transcriptome sequencing of carefully identified mouse TEC subpopulations, we discovered a program of PGE that is common between medullary (m) and cortical TEC, further elaborated in mTEC, and completed in mature mTEC expressing the autoimmune regulator gene (*Aire*). TEC populations are capable of expressing up to 19,293 protein-coding genes, the highest number of genes known to be expressed in any cell type. Remarkably, in mouse mTEC, *Aire* expression alone positively regulates 3980 tissue-restricted genes. Notably, the tissue specificities of these genes include known targets of autoimmunity in human *AIRE* deficiency. Led by the observation that genes induced by *Aire* expression are generally characterized by a repressive chromatin state in somatic tissues, we found these genes to be strongly associated with H3K27me3 marks in mTEC. Our findings are consistent with AIRE targeting and inducing the promiscuous expression of genes previously epigenetically silenced by Polycomb group proteins. Comparison of the transcriptomes of 174 single mTEC indicates that genes induced by *Aire* expression are transcribed stochastically at low cell frequency. Furthermore, when present, *Aire* expression-dependent transcript levels were 16-fold higher, on average, in individual TEC than in the mTEC population.

T cell-mediated responses are essential in providing protective immunity but depend on an acquired ability to discriminate between foreign and self-antigens. This capacity is instructed during T cell development in the thymus by populations of cortical and medullary thymic epithelial cells (TEC) (Holländer et al. 2006). Cortical TEC (cTEC) provide signals that commit hematopoietic precursors to a T cell fate and positively select immature T cells (thymocytes) that express a functionally competent T cell receptor (TCR) for further differentiation. Following migration to the medulla, thymocytes are further selected by medullary TEC (mTEC). T cells with a high affinity TCR for self-antigens are deleted whereas those with a TCR of intermediate affinity are diverted to a regulatory (T_reg_) fate. These mechanisms of clonal deletion and clonal diversion ensure that only thymocytes with low self-affinity will differentiate into effector T cells (T_eff_) and hence establish central tolerance of self.

In order to assess T cell self-reactivity, cTEC and mTEC express and present hundreds of peripheral tissue-restricted antigens (TRA) (Derbinski and Kyewski 2010; Anderson and Takahama 2012). The diverse expression of TRA by TEC contrasts with the tight spatio-temporal control of gene expression observed in peripheral tissues during pre- and post-natal development and is conceptually referred to as promiscuous gene expression (PGE). PGE is believed to be broader in mTEC than cTEC, and is positively correlated with mTEC differentiation (Derbinski et al. 2005). Importantly, estimates that mTEC promiscuously express up to 3000 TRA also implied that many thousands of additional genes would not be expressed in TEC and consequently not employed for the screening of T cells reactive to self (Kyewski and Derbinski 2004).

Currently, the relative contributions of TEC, migratory dendritic cells, and mechanisms of peripheral tolerance to the avoidance of autoimmunity are poorly understood (Bonasio et al. 2006; Hadeiba et al. 2012; Xing and Hogquist 2012). It is also unclear whether the TCR repertoire of thymocytes needs to be selected against all or, alternatively, against only a specific subset of self-antigens in order to effectively establish central tolerance. To answer these questions, it is essential to first determine the identity of all self-antigens promiscuously expressed by TEC because this would define the extent and resolution of self-tolerance mediated by these cells. Similarly, analysis of the nature of PGE in cTEC would be crucial for the understanding of the initial positive selection of thymocytes and may also be relevant for understanding their post-thymic homeostasis.

Distinction of PGE in TEC from the transcriptional programs in peripheral tissues (Villaseñor et al. 2008) appears to depend for some TRA on an as yet only incompletely understood mechanism involving the nuclear protein Autoimmune regulator (AIRE) (for review, see Mathis and Benoist 2009). This mechanism is as ancient as the adaptive immune system itself, because *Aire* has now also been identified in all classes of jawed vertebrate following its recent discovery in cartilaginous fish (Venkatesh et al. 2014). In humans, *AIRE* is primarily expressed in mTEC and its loss-of-function mutations cause the autoimmune polyendocrine syndrome type-1 (APS-1; OMIM #240300), which is marked by the survival and thymic export of self-reactive T_eff_ cells (Mathis and Benoist 2009). Consequently, the syndrome is characterized by severe organ-specific autoimmunity typically affecting parathyroid chief cells, steroidogenic cells of the adrenal cortex, pancreatic β-cells, gastric parietal cells, skin melanocytes, hepatocytes, gonads, and the lung (Shikama et al. 2009; Shum et al. 2013).

Within the mTEC lineage, the role of *Aire* in facilitating PGE has not yet been precisely assessed (Anderson et al. 2002). Although microarray analyses of mature (MHCII^hi^) mTEC revealed 1343 genes regulated by *Aire* expression that represent many tissues of the body (Venanzi et al. 2007), these approaches are compromised both by the heterogeneity of mature mTEC, of which only half express *Aire*, and by a limitation of microarray technology, specifically that this method is prone to cross-hybridization artifacts that make confident detection of lowly expressed genes difficult (Gardner et al. 2008; Mortazavi et al. 2008). Furthermore, while limited analyses have argued that AIRE acts stochastically (Derbinski et al. 2008; Villaseñor et al. 2008), a genome-wide analysis of both PGE extent and diversity at the single-cell level has not been previously undertaken.

A complete mapping of the PGE program in individual TEC subsets should yield insights into molecular mechanisms that underlie this process. Presently, these remain largely unknown with the notable exception of the involvement of AIRE which interacts with proteins involved in chromatin structure, DNA damage response, gene transcription, and RNA processing (Abramson et al. 2010; Gaetani et al. 2012). Currently, AIRE is understood to act by recruiting the positive transcription elongation factor b (P-TEFb) to inactive genes at which stalled RNA polymerase II (Pol II) is already present (Zumer et al. 2013). The functions of the various domains of AIRE have been the focus of several studies, with the second plant homeodomain (PHD2) and the C-terminal domain having been shown to be critical for the transactivation of target genes (Meloni et al. 2008).

The mechanism by which AIRE is targeted to particular genomic locations is only incompletely understood but is thought to involve the PHD1 domain of AIRE directly recognizing unmethylated H3K4 (H3K4me0), a histone mark characteristic of inactive promoters (Koh et al. 2008; Org et al. 2008). However, while this interaction is crucial for targeting in vitro (Zumer et al. 2012), abolition of this interaction in vivo only dampened the impact of AIRE without affecting its transcriptional range (Koh et al. 2010). Recently, a role for DNA-dependent protein kinase (PRKDC) in the targeting of AIRE to inactive promoters has been described (Zumer et al. 2012), suggesting that other known AIRE interaction partners (Abramson et al. 2010; Gaetani et al. 2012) may also play important roles in this process.

To address these issues, we performed deep transcriptome sequencing to reveal the extent and character of PGE within distinct TEC subpopulations and within single mature TEC. The digital nature and increased dynamic range of RNA-seq permit a more precise assessment of the number of genes expressed by particular cell types and tissues. While RNA-seq has shown tissues to express on average as few as 8000 or as many as ∼13,500 genes (Ramsköld et al. 2009), this technology has not yet been applied to specific TEC subsets (St-Pierre et al. 2013) or individual TEC. Here, we analyze PGE in cTEC and specific mTEC subsets, precisely identifying the pattern of genes expressed by either all or individual TEC subsets and relate these results to thymic selection. Based on the results of in vivo chromatin state analysis, we suggest that AIRE targets and induces the expression of genes epigenetically silenced by Polycomb group proteins. Analysis of single mTEC transcriptomes revealed *Aire* expression-regulated gene transcription to be an apparently stochastic, low frequency phenomenon. When transcribed, genes regulated by *Aire* expression have substantially higher levels in individual cells than those indicated by population analyses.

## Results

### Isolation of *Aire*-positive and -negative TEC subpopulations

**Figure 1.**
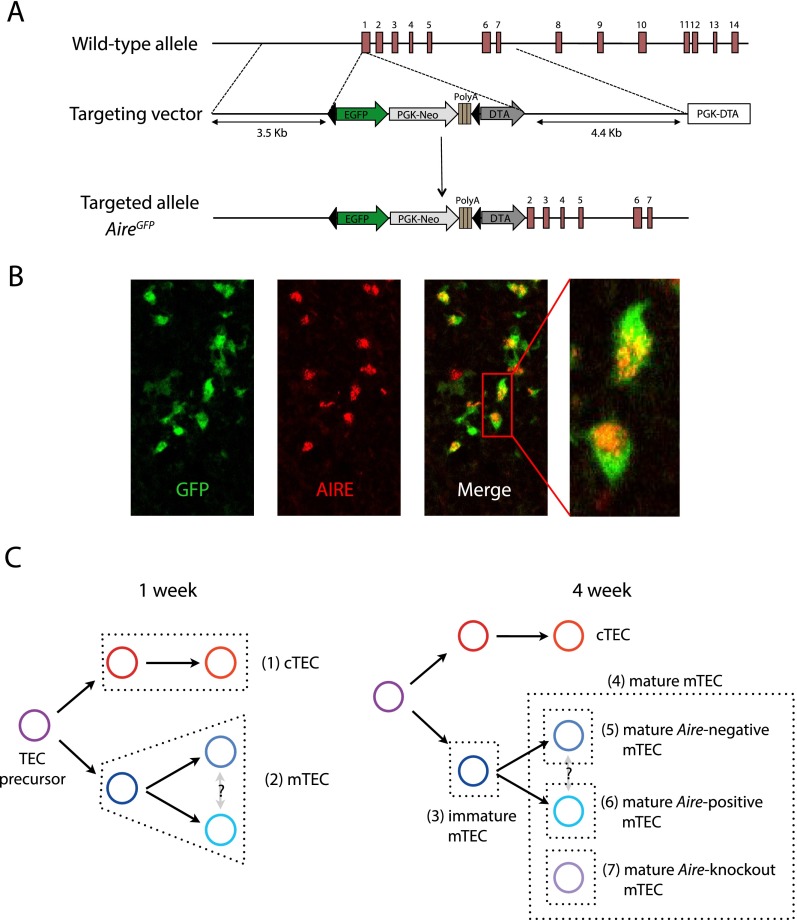
Generation of *Aire*^*GFP/+*^ mice. (*A*) The genomic *Aire* locus (*top*), with the targeting construct (*middle*), and the targeted locus (*bottom*). Red rectangles with numbers indicate exons and black triangles indicate *loxP* sites. The PGK neo cassette in the targeting construct is followed by triple poly(A) signals to prevent further transcriptional elongation. (*B*) Immunofluorescence analysis of thymus sections of *Aire*^*GFP/+*^ mice for GFP (green) and AIRE (red). (*C*) The basic scheme of TEC differentiation and the identification of individual TEC populations. Seven (1–7) distinct TEC populations were sorted from thymic tissue isolated from wild-type C57BL/6, *Aire*^*GFP/+*^, and *Aire*^*GFP/GFP*^ mice (Supplemental Table 1; Supplemental Fig. 1). To ensure that the distinct mTEC subsets had fully differentiated during post-natal maturation in the presence of regular thymopoiesis and that these cells were sufficiently abundant for analysis (Irla et al. 2008), we collected the diverse mTEC populations from 4-wk-old animals, whereas cTEC and total mTEC were sorted from mice at 1 wk of age.

The subset of mature mTEC that express *Aire* cannot be distinguished by known specific cell surface markers (Anderson et al. 2002; Mathis and Benoist 2009). To specifically assay the transcriptomes of *Aire*-positive and -negative mTEC, we therefore generated a novel mouse line (designated *Aire*^*GFP/+*^) that expresses the enhanced green fluorescent protein (GFP) under the transcriptional control of the endogenous *Aire* locus (Fig. 1A). In *Aire*^*GFP/+*^ mice, GFP expression was restricted to mTEC that mostly co-expressed *Aire* as identified by immunohistochemistry (Fig. 1B) and by high cell surface concentrations of MHC class II molecules as verified by flow cytometry (Supplemental Figs. 1, 2A). We inspected residual transcription from the modified *Aire* locus and confirmed it to be largely limited to the unmodified 5′ UTR (Supplemental Fig. 2B). These results demonstrated the utility of *Aire*^*GFP/+*^ mice in enabling the isolation of distinct mature mTEC subsets that differ in their *Aire* expression.

### Transcriptomic analysis of TEC reveals the full extent of PGE

**Figure 2.**
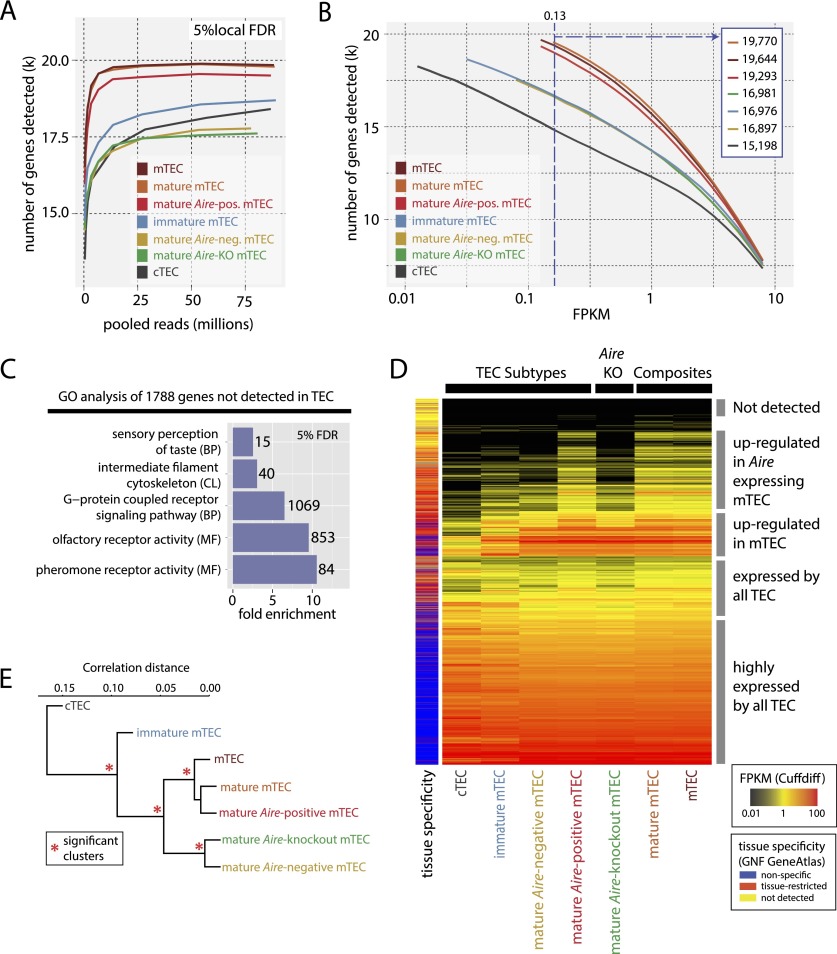
RNA-seq analysis reveals the full extent of PGE in TEC. (*A*) The number of genes detected in each FACS sorted TEC population at a local FDR of 5% (see Methods; Supplemental Fig. 3; Supplemental Table 2) as a function of read depth (pooled replicates) indicates that read depth was not limiting for most of the TEC populations. (*B*) The number of genes detected in the TEC populations at different FPKM thresholds. The *left*-hand start of the solid lines indicates the expression level that corresponds to a local FDR of 5% for a given TEC population (see also Supplemental Fig. 3C). The vertical blue dashed line indicates the FPKM at which genes can be reliably detected in all TEC types (numbers of genes detected at this threshold are shown in *inset*). (*C*) Selected GO categories enriched in genes not detected in any TEC population reveal a striking enrichment for odorant receptors (Supplemental Fig. 5). (CL) Cellular location; (BP) biological process; (MF) molecular function. (*D*) Hierarchical clustering of the expression levels of all protein-coding genes in the TEC populations reveals three distinct strata of PGE. The color key to the *left* of the heatmap indicates the tissue specificity of genes in the GNF GeneAtlas according to the dynamic step method (Methods; Supplemental Fig. 6). (*E*) Hierarchical clustering of the TEC populations by gene expression correlation distance reveals four significant clusters (red asterisks, *P* > 0.95).

We sequenced the transcriptome of two biological replicates for each of seven FACS-sorted TEC populations (Methods; Fig. 1C; Supplemental Fig. 1; Supplemental Table 1) yielding an average of 39.5 (31.3–42.9) million mapping reads per sample. Employing a novel local false discovery rate (FDR) approach that enables a per-gene estimate of expression probability (Supplemental Fig. 3), we discovered that the assayed cTEC and mTEC populations expressed 84% and 89%, respectively, of Ensembl (Flicek et al. 2013) protein-coding genes (Fig. 2A; Supplemental Table 2). Comparable results were obtained using a previously described global FDR approach (Supplemental Fig. 3E; Ramsköld et al. 2009). Notably, the expression of many genes was much stronger in mTEC than in cTEC (Fig. 2B). Across a range of FPKM thresholds, the TEC subtypes clearly separated into three groups based on the numbers of genes they expressed: (i) mature mTEC expressing *Aire*, (ii) immature mTEC and mature mTEC lacking *Aire* expression, and (iii) cTEC (Fig. 2B).

At an FPKM threshold of 0.13, at which genes can be reliably detected in all of the TEC populations assayed (dashed blue line, Fig. 2B), the mature *Aire*-positive mTEC population expressed 87% (19,293) of Ensembl protein-coding genes. To our knowledge, this is the highest proportion of genes yet found to be expressed in any cell type. By comparison, immature mTEC and mature mTEC subtypes lacking *Aire* expression transcribed fewer genes at the same threshold—on average 76% (16,951) while cTEC only expressed 68% (15,198) genes. An alternative differential expression approach based on count data yielded similar conclusions (Supplemental Fig. 4A,B; Supplemental Table 3).

These results reveal the breadth of gene expression within the TEC lineages including the presence of an extraordinary near-complete transcriptional program in *Aire*-positive mTEC. We next characterized the 1788 genes without detectable transcription at a 5% local FDR threshold in any of the TEC populations. As these genes will not contribute to the mixture of self-peptides shaping the self-tolerant TCR repertoire, their identification may relate to targets for tissue-specific autoimmunity. Gene ontology (GO) analysis revealed a strong enrichment of odorant receptors among these genes (Fig. 2C; Supplemental Fig. 5), and inspection revealed them to be comprised largely of olfactory receptors (882 genes, 49%), vomeronasal receptors (276 genes, 15%), and genes of unknown function (398, 22%) that are annotated as RIKEN clones or unannotated Ensembl gene models (a set expected to contain pseudogenes and noncoding RNAs wrongly assigned as encoding proteins). Overall, approximately half of all olfactory and vomeronasal receptor genes showed weak or sub-detection threshold expression (Supplemental Fig. 5C). Therefore, our results revealed that mTEC can express virtually the entire repertoire of known protein-coding genes, with the notable exception of about a thousand odorant receptor genes, and thus may provide a near comprehensive basis for the screening of the randomly generated TCR repertoire for self-reactivity.

### PGE by TEC is stratified into three major tiers

We next established the extent of PGE in individual TEC populations by examining the expression of TRAs. Here, we took advantage of version 3 of the microarray-based GNF Mouse GeneAtlas (GNF GeneAtlas) (Lattin et al. 2008). First, we hierarchically clustered the 64 physiological samples (excluding the thymus) by correlation distance into 35 sample groups to reduce representation bias (Supplemental Fig. 6A). We then identified genes as being tissue restricted if they showed substantively higher expression in one to five of these tissue groups by utilizing a novel dynamic step method that shows a greater sensitivity and specificity than a previous simple threshold approach (Supplemental Methods; Supplemental Fig. 6; Supplemental Table 2; Gardner et al. 2008).

Hierarchical clustering of levels of all protein-coding genes expressed in TEC revealed three major groups of tissue-restricted genes: (i) those commonly expressed by all of the TEC populations; (ii) those expressed more highly by mTEC; and (iii) those up-regulated in *Aire*-expressing mature mTEC (Fig. 2D; Supplemental Fig. 4C,D). Above an FPKM threshold of 0.13 we found 3378 TRAs to be commonly expressed by cTEC, mature *Aire*-negative mTEC, and mature *Aire*-positive mTEC, 4623 TRAs to be expressed by both *Aire*-negative and *Aire*-positive mature mTEC, and 5970 TRAs to be expressed in mature *Aire*-positive mTEC (Supplemental Fig. 4D). PGE is thus stratified into three major tiers comprising a basic program common to all TEC subtypes that is further elaborated in mTEC and completed in *Aire*-expressing mature mTEC.

### Pathway analysis of three major TEC types

The molecular features of TEC subtypes and their developmental relationships are ill-defined and are the subject of debate (for review, see Alves et al. 2014). Based on the hierarchical clustering of gene expression correlation, we found the TEC populations to fall into four significant clusters comprised of cTEC, immature mTEC, mature mTEC lacking *Aire* expression, and mature *Aire*-expressing mTEC (Fig. 2E). However, because these relationships may be obscured by PGE we reexamined TEC clustering after excluding tissue-restricted genes and genes induced by *Aire* expression. The TEC populations now clustered into only three significant groups (Supplemental Fig. 7A) comprising cTEC, immature mTEC, and mature mTEC, suggesting that these cell populations represent three fundamentally distinct TEC identities. Consistent with this finding, mature mTEC with an inactive *Aire* locus yield a near identical gene profile to those with an active *Aire* locus (Supplemental Fig. 8D).

We next compared the transcriptomes of these three TEC types, identifying biological pathways significantly enriched among genes differentially expressed between mature *Aire*-negative mTEC, immature mTEC, and cTEC (Supplemental Tables 3, 4; Supplemental Figs. 7, 8A–C). This approach revealed pathways previously known to be relevant for TEC biology (Supplemental Fig. 7B; Supplemental Table 4), including the JAK-STAT and NF-kappa B-signaling pathways (Supplemental Fig. 7C,D). In a complementary analysis, we sought genes that were highly expressed in a given TEC subtype (>10 FPKM) at a level at least twice that observed in any other TEC subtype. This recovered known TEC marker genes such as *Ctsl*, *Dll4*, *Psmb11* (also known as *beta5t*), and *Cxcl12* which all identify cTEC. Furthermore, this analysis revealed genes that were not known previously to identify individual TEC types (Supplemental Fig. 8F; Supplemental Table 5). The delineation of pathways and genes characteristic of specific TEC types offers important insights into the specification of these populations.

### *Aire* secures the promiscuous expression of thousands of TRAs

**Figure 3.**
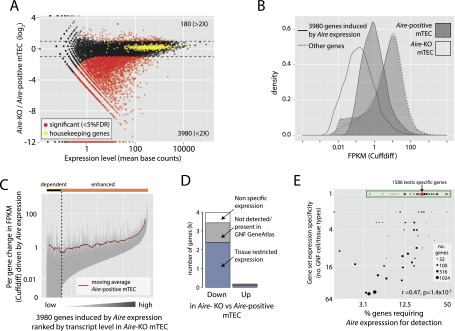
*Aire* expression positively regulates a large set of tissue-restricted genes. (*A*) Genes differentially expressed between mature *Aire*-positive mTEC and mature *Aire*-KO mTEC (<5% FDR, more than twofold). A set of 474 housekeeping genes (de Jonge et al. 2007) showed little change in expression, indicating the absence of a systematic bias (yellow points). (*B*) At the population level, *Aire* expression elevates target gene transcription to a median FPKM of 1. (*C*) *Aire* expression differentially up-regulates individual target genes. Each gray vertical line represents the change in FPKM of a single gene between mature *Aire*-KO mTEC and mature *Aire*-positive mTEC. Genes are ordered by increasing expression in mature *Aire*-KO mTEC on the *x*-axis, being either dependent on or enhanced by *Aire* expression. The red line represents the moving average of FPKM in mature *Aire*-positive mTEC. (*D*) Genes induced by *Aire* expression are tissue-restricted in transcription. Tissue-restricted genes were identified from the GNF GeneAtlas using the dynamic step method (Methods; Supplemental Fig. 6). (*E*) Degree of tissue restriction is positively correlated with the requirement for *Aire* expression. The fraction of genes requiring *Aire* expression for detection was assessed for sets of genes restricted in expression to all possible branches, nodes, and leafs of the GNF GeneAtlas sample clustering (Supplemental Fig. 6A). Only gene sets with at least 10 members are shown. The red dot indicates 1586 genes restricted in expression to testis, a tissue with a transcriptome of abnormally high complexity (Ramsköld et al. 2009) that is known to express *Aire* and to undertake PGE (Schaller et al. 2008).

In order to elucidate more precisely the role of *Aire* in PGE, we identified genes differentially expressed between mature *Aire*-positive mTEC and mature *Aire*-knockout (*Aire*-KO) mTEC. *Aire* expression positively regulated (induced) 3980 Ensembl protein-coding genes, but negatively regulated only 180 genes (more than twofold, 5% FDR) (Fig. 3A; Supplemental Table 3). Of the genes up-regulated by *Aire* expression, 594 (15%) were entirely dependent on *Aire* expression (i.e., their transcripts were not detected in *Aire*-KO mTEC), whereas *Aire* expression elevated the transcription of 3386 genes (85%) (Fig. 3C).

Given the diverse clinical presentation of APS-1, we next examined the tissue specificity of genes regulated by *Aire* expression, finding the large majority (86%) of induced genes to be either tissue-restricted or not detected in the GNF GeneAtlas (Fig. 3D). Furthermore, analysis of exclusive sets of genes with varying degrees of expression breadth across the GNF GeneAtlas uncovered a positive relationship between the fraction of genes in a set requiring *Aire* expression for detection and the set’s tissue-expression specificity (r = 0.47, *P* = 1.4 × 10^−5^) (Fig. 3E). *Aire* expression therefore governs the promiscuous expression of a set of TRA that is substantially greater in extent than previously suspected, and it elevates the expression of these genes to a typical minimum level of 1 FPKM in the mature *Aire*-expressing mTEC population.

### The tissue specificity of genes regulated by *Aire* expression includes organs affected in APS-1

**Figure 4.**
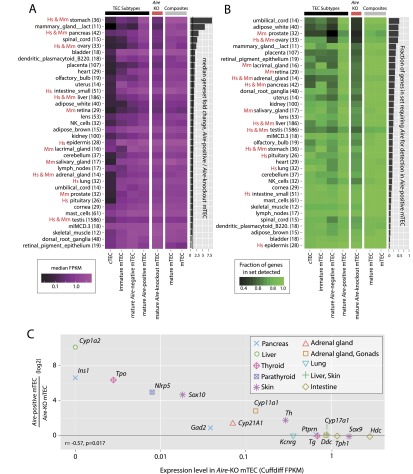
Requirement for *Aire* reflects known *AIRE* deficiency pathologies. (*A*) The median expression level (FPKM) of sets of genes restricted in expression to single physiological samples (excluding the thymus) of the GNF GeneAtlas (based on dynamic step criteria; see Methods; Supplemental Fig. 6) (gene numbers indicated in parentheses) for each TEC population. Gene sets are sorted by the fold change in median expression level between mature *Aire*-positive and mature *Aire*-KO mTEC (accompanying bar chart). In both *A* and *B*, gene sets representing organs affected by *AIRE* deficiency in APS-1 (“Hs”) and the corresponding mouse model (“Mm”) are indicated. (*B*) The fraction of the same sets of genes that are detectable (<5% local FDR) in each TEC population (see Methods; Supplemental Fig. 3); gene sets are sorted by the increase in the fraction of these genes detected in mature *Aire*-positive mTEC compared to *Aire*-KO TEC. (*C*) Relative expression of known APS-1 autoantigens in mature *Aire*-positive wild-type and knockout TEC (Shikama et al. 2009). Induction by *Aire* expression is significantly negatively correlated with the transcriptional level of these genes in mature *Aire*-KO mTEC.

Given the organ-specific nature of autoimmunity in APS-1 patients, we next considered transcripts of sets of genes whose expression is restricted to single physiological GNF GeneAtlas samples (green box, Fig. 3E) assessing both their expression level (Fig. 4A) and presence in each TEC population (Fig. 4B). It is notable that genes whose expression is restricted to tissues that are commonly affected in human *AIRE* deficiency or in analogous mouse models (Shikama et al. 2009; Kurisaki et al. 2013) showed especially strong dependence on *Aire* expression (denoted Hs and Mm, respectively) (Fig. 4A,B).

The parathyroid and adrenal glands are the signature sites of autoimmunity in APS-1. While the parathyroid is not represented in the GNF GeneAtlas, 14 genes restricted to the adrenal gland GNF GeneAtlas sample showed a relatively high requirement for *Aire* expression for transcription in mTEC (Fig. 4B). Genes with transcripts restricted to the reproductive system were also among those displaying the highest degree of dependency on *Aire* expression (Fig. 4B). This reliance is reflected in the clinical observation that premature ovarian failure, oophoritis, primary testicular failures and autoantibodies against the steroidogenic enzymes, 21-hydroxylase (CYP21A1), steroid 17-alpha hydroxylase (CYP17A1) and P450scc (CYP11A1) constitute common autoimmune features of APS-1 (Betterle and Zanchetta 2003). We also observed tissues whose antigens were largely induced by *Aire* expression but that have thus far not been identified as targets of APS-1-related pathologies (Fig. 4A,B) such as several female reproductive system tissues (Perheentupa 2006).

Only half of the mouse orthologs of known APS-1-relevant autoantigens showed a clear dependence on *Aire* expression, with dependency being largely predicted by the level of ortholog transcription in *Aire*-KO mTEC (Fig. 4C). Notably, however, the spectrum of tissues represented by the APS-1 autoantigens was largely recapitulated among the affected mouse orthologs which, together with the broader analyses (Fig. 4A,B), indicate a strongly conserved role for *Aire* between these two species.

### Genes regulated by *Aire* expression have a repressive chromatin state in somatic tissues

To gain insight into the mechanism by which AIRE is targeted to particular genomic loci, we inspected features of genes that are positively regulated by *Aire* expression. While the genomic locations and structures of these genes do not show obvious architectural differences (data not shown), we reasoned that they may possess a silenced chromatin state that is common to TEC and most somatic cell types. To test this idea we used data from the Mouse ENCODE Project (The Mouse ENCODE Consortium et al. 2012) to assess the presence of chromatin modifications at gene transcriptional start sites (TSS).

**Figure 5.**
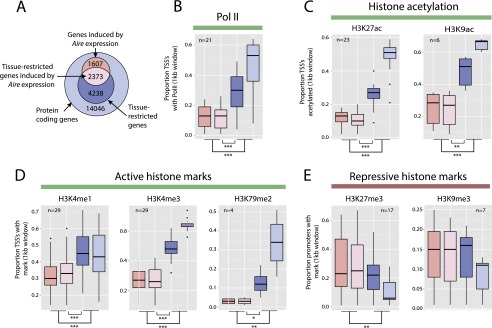
Genes induced by *Aire* expression are characterized by a repressive chromatin state in somatic tissues. (*A*) Genes were divided into sets comprising *Aire* expression-induced (light pink) or *Aire* expression-independent TRAs (dark blue), other genes induced by *Aire* expression (dark pink), and all other genes (light blue). The proportion of genes in each of these sets with TSS overlapping Mouse ENCODE ChIP-seq peaks in various tissue and cell types was assessed. (*B*) Box and whisker plots show the distribution of proportions of the four gene sets (see panel *A*) overlapping RNA polymerase II (Pol II) ChIP-seq calls from 21 Mouse ENCODE samples. The TSS of tissue-restricted genes induced by *Aire* expression overlap significantly less frequently with Pol II binding sites than those of other TRAs. A similar pattern was observed for histone acetylation (*C*) and active histone marks (*D*). In contrast, the TSS of genes induced by *Aire* expression show significantly greater overlap with H3K27me3 across 17 Mouse ENCODE samples (*E*). The *n*-values represent the number of Mouse ENCODE samples analyzed. (*) *P* < 0.05, (**) *P* < 0.01, (***) *P* < 0.001, using the Mann-Whitney *U*-test. Colors as in *A*.

Compared to those of other TRAs, the TSS of genes induced by *Aire* expression were broadly and significantly depleted in Pol II binding (Fig. 5B), in promoter acetylation (Fig. 5C) and in active histone marks (Fig. 5D). In contrast, the TSS of genes up-regulated by *Aire* expression showed a significant enrichment for the repressive histone mark H3K27me3 (Fig. 5E), a mark of particular interest given the established role of Polycomb group proteins in lineage-specific gene silencing. Overall, the epigenetic landscape we observed is consistent with the known ability of AIRE to recognize H3K4me0 and suggested a role for AIRE in derepressing genes silenced by Polycomb group proteins.

### Genes regulated by *Aire* expression are associated with the mark of Polycomb silencing

**Figure 6.**
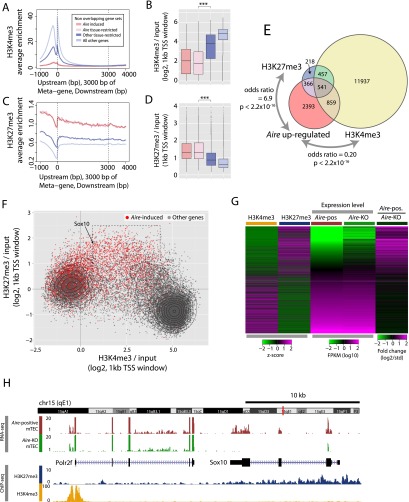
*Aire* expression is associated with transcription of Polycomb silenced genes in mTEC. (*A*) Metagene profiles of the average normalized enrichment of H3K4me3 against input for sets of genes distinguished by *Aire* dependence and tissue specificity. (*B*) Boxplots of the median enrichment of H3K4me3 at the TSS of these sets of genes. (*C*,*D*) The results of the corresponding analysis for H3K27me3 marks. In *B* and *D*, *** indicates a significance level of *P* < 0.001 using the Mann-Whitney *U*-test. (*E*) The association of genes up-regulated by *Aire* expression (>2×, FDR < 0.05, *Aire*-positive vs. *Aire*-knockout mTEC) with genes whose TSS (1-kb centered windows) showed an average (*n* = 2) twofold or greater enrichment for H3K27me3 or H3K4me3 marks over input. Stated *P*-values were calculated using Fisher’s exact test. (*F*) Enrichment of H3K4me3 and H3K27me3, respectively, for the TSS of all protein-coding genes. Those induced by *Aire* expression are highlighted in red. The dashed box highlights a subset of genes induced by *Aire* expression whose TSS show enrichments for both modifications. (*G*) Genes induced by *Aire* expression are generally weakly transcribed in *Aire-*KO mTEC and have relatively low H3K4me3 enrichment scores and relatively high H3K27me3 enrichment scores. The heatmap shows all genes ordered by their ratio of expression in mature *Aire*-positive and knockout TEC. (*H*) The chromatin state and expression of APS-1 autoantigen ortholog *Sox10* and *Polr2f* encoding a RNA Pol II subunit in mature mTEC.

To test whether AIRE positively regulates the expression of genes held in a repressive chromatin state characterized by the presence of H3K27 trimethylation, we analyzed the epigenetic state of mature mTEC in vivo using chromatin immunoprecipitation (ChIP) followed by massively parallel DNA sequencing (ChIP-seq). Metagene analyses of uniquely mapped ChIP-seq reads revealed that genes positively controlled by *Aire* expression in mTEC tend to be held in a repressed state: There is a striking and significant reduction of H3K4me3 (Fig. 6A,B), a marker of actively transcribed genes, and a corresponding significant enrichment for H3K27me3 (Fig. 6C,D) at their TSS. Strikingly, 1582 genes that showed at least twofold enrichment for H3K27me3 were highly enriched for annotations associated with organismal development (Supplemental Fig. 9) and were strongly associated with regulation by *Aire* expression (odds ratio 6.9, *P* < 2.2 × 10^−16^) (Fig. 6E).

More generally, the TSS of all genes separated either into an active state characterized by high levels of H3K4me3 and an absence of H3K27me3, or into a converse repressive state (Fig. 6F). The TSS of genes up-regulated by *Aire* expression fell mostly into the repressive state, although a subset showed higher, intermediate levels of H3K4me3 while also being marked by relatively high levels of H3K27me3 (dashed box, Fig. 6F). While there was a clear negative correlation between the enrichment of the two marks for most TSS, the two marks showed a significant positive correlation (r = 0.21, *P* < 2.2 × 10^−16^) at the TSS of genes up-regulated by *Aire* expression.

Overall, genes induced by *Aire* expression were distinguished by higher levels of H3K27me3 and lower levels of H3K4me3 in the mature mTEC population, and by low expression levels in *Aire*-KO mTEC (Fig. 6G). By way of illustration, the *AIRE*-regulated APS-1 autoantigen ortholog *Sox10* was barely expressed in *Aire*-KO mTEC and was marked by H3K27me3 but appeared devoid of H3K4me3 (Fig. 6H). Immediately adjacent on chromosome 15 (but transcribed in the opposing orientation) lies the *Aire*-insensitive gene for the ubiquitous RNA polymerase II subunit *Polr2f.* This gene was strongly marked by H3K4me3 at its TSS but showed negligible enrichment for H3K27me3 (Fig. 6H; for further examples, see Supplemental Fig. 10). In summary, these population level observations suggest that H3K27me3 is associated with the recruitment of AIRE to target loci even in the presence of H3K4me3.

### Genes regulated by *Aire* expression are stochastically transcribed at low frequency in individual mTEC

**Figure 7.**
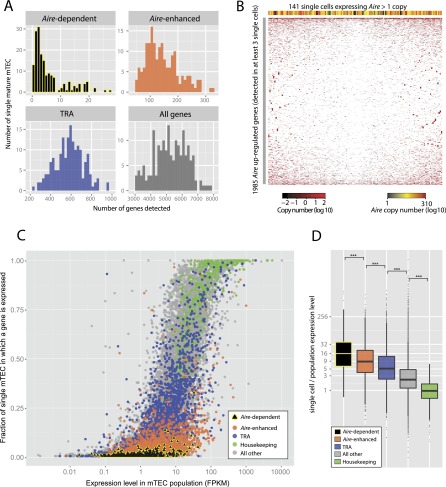
Transcriptomic analysis of promiscuous gene expression in single TEC. (*A*) Single mature mTEC tend to express few genes that are dependent on or enhanced by *Aire* expression (as defined in Fig. 3C). The histograms show the number of genes detected in 174 single mature mTEC that expressed >3000 protein-coding genes. (*B*) No discernible clustering is evident from the hierarchical clustering (with optimized leaf ordering) of 141 single *Aire*-expressing mature mTEC (columns) and 1985 genes up-regulated by *Aire* expression (rows) detected in at least three of these single cells. The colored bar *above* the plot indicates the single-cell expression level of *Aire*. (*C*) Genes dependent on *Aire* expression are transcribed less frequently in single mature mTEC than are other genes. The scatter plot shows the fraction of single mature mTEC that express any given gene against the expression level of that gene determined from the mature mTEC population. (*D*) When transcribed in single mTEC, genes dependent on *Aire* expression tend to be present at a level 16-fold higher than that indicated by the population average. Before calculating the relative expression levels, single-cell gene expression levels were globally normalized against population values using a linear model. (***) *P* < 1 × 10^−14^, estimated using the Mann-Whitney *U*-test.

To compare gene expression in the mature mTEC population and in single cells we sequenced the individual transcriptomes of 190 mature mTEC. Gene expression was quantitated using spike-ins (Supplemental Fig. 11A,B; External RNA Controls Consortium 2005) and 174 of these cells were further analyzed based on their expression of more than 3000 protein-coding genes (Fig. 7A). Gene expression across the 174 cells showed good correspondence with that observed at the population level, with the mean expression levels of 18,945 genes detectable at the single-cell level (Supplemental Fig. 11C) showing good correlation with those from the mature mTEC population (Spearman’s r = 0.83, *P* < 2.2 × 10^−16^) (Supplemental Fig. 12A). In individual mature mTEC we detected the transcription of an average of 5262 genes, including 0–27 *Aire* expression-dependent (mean = 3), 46–322 (mean = 144) *Aire* expression enhanced, and 241–969 (mean = 581) *Aire* expression independent TRA (Figs. 3C, 7A). Numbers of genes detected per cell are likely underestimates due to the limited detection sensitivity of lowly expressed genes at the single-cell level (assessed in Supplemental Fig. 11B; Marinov et al. 2014). The number of genes transcribed by a cell and the per-cell *Aire* expression level were significantly correlated (Spearman correlation r = 0.40, *P* = 4.24 × 10^−8^) (Supplemental Fig. 12B,C).

Next, we assessed whether genes up-regulated by *Aire* expression were co-expressed non-randomly across 141 *Aire-*positive single TEC. Hierarchical clustering analysis of these genes and cells, however, failed to reveal clear clusters (Fig. 7B). While it is conceivable that nonstochasticity might be apparent with substantially more cells, these data, together with previous analyses of a small number of genes across an order of magnitude more TECs (Derbinski et al. 2008; Villaseñor et al. 2008), provide strong support for the hypothesis that genes up-regulated by AIRE are stochastically expressed in single TEC.

Finally, we sought to establish the frequency and level of self-antigen expression in TEC. We found that the frequency of self-antigen expression in mature mTEC is consistent with previously reported qPCR data (Supplemental Fig. 12D; Derbinski et al. 2008; Villaseñor et al. 2008). Furthermore, when plotted against expression level in the mature mTEC population, the proportion of TEC in which a gene is expressed demonstrated an overall sigmoidal relationship (Fig. 7C). Housekeeping genes showed high population expression and were typically detected in at least half (median 84%) of all single mTEC. In contrast, genes entirely dependent on *Aire* expression for their transcription were detected in a very small fraction (median 1.1%) of mature mTEC regardless of their population expression level. Genes enhanced by *Aire* expression were also infrequently transcribed (median 2.3%) while *Aire*-independent TRA were more commonly observed (median 9.2%) but less so than all other genes (median 33.3%) in single mature mTEC (Supplemental Fig. 12E). Notably, when present, genes dependent on *Aire* expression were much more highly transcribed (median 16-fold) in single cells than in the population (Fig. 7D) just as, to a lesser extent, were genes enhanced by *Aire* expression (median ninefold) and non-*Aire* induced TRA (median fivefold) (Fig. 7D). Self-antigens that tend to be lowly expressed at the population level are thus highly but infrequently transcribed in individual TEC.

## Discussion

The initial positive selection of the randomly generated TCR repertoire by cTEC critically depends on the expression, processing, and presentation of a diverse set of self-peptides (Starr et al. 2003; Klein et al. 2009; Viret et al. 2011). The repertoire of self-peptides presented by cTEC is also of interest because these cells contribute to negative selection (Ahn et al. 2008; McCaughtry et al. 2008; Stritesky et al. 2013), and the generation of T_reg_ (Liston et al. 2008). Moreover, education on self-peptides presented by cTEC is essential for maintaining T cell survival and homeostatic expansion in the periphery (Ernst et al. 1999). While we detect similar numbers of expressed genes in cTEC and in *Aire*-negative mTEC (Fig. 2B), the promiscuous expression of tissue-specific antigens is notably weaker in the cTEC population (Figs. 2D, 4A), which may impact on the efficiency of cTEC-mediated selection. Notwithstanding, and in addition to providing the basis for the positive selection of a diverse array of TCRs, a broad molecular representation of thousands of self-antigens by cTEC likely establishes a front line for both the clonal deletion and diversion of self-reactive T cells.

For central tolerance to be effective, T cells must be challenged in the thymus with a complex array of peptides, which foretells the ubiquitous and tissue-restricted self-antigens that they will encounter at any point in their lifetime monitoring somatic cells in the periphery. Our results show for the first time that mature *Aire*-positive mTEC are capable of transcribing the vast majority of the protein-coding genome in order to meet this need. Most of these genes are lowly expressed at the population level, disguising infrequent but relatively higher expression at the single-cell level. Hence, PGE likely results in the availability of sufficient TRA to be efficiently presented for thymic selection. Together, our population and single-cell analyses suggest that TEC alone may suffice for the establishment of central tolerance that is broadly comprehensive rather than selective in nature (the contributions by thymic dendritic cells in this process have, however, not been addressed by our studies). Furthermore, the relatively low number of genes dependent on *Aire* expression that we observe to be transcribed in single thymic epithelia provides a natural explanation for the persistent need for a large *Aire-*positive mTEC population to efficiently effect central tolerance.

In mature mTEC, expression of *Aire* secures the promiscuous transcription of an unprecedented number of TRAs. These genes are typically undetectable or lowly transcribed in the absence of *Aire* expression but in its presence tend to exceed a minimum of ∼1 FPKM at the population level, and are substantially more highly expressed in the single cells in which they are present. We show here for the first time that, at genome scale, genes regulated by *Aire* expression have a sporadic distribution at the single-cell level. We thus are led to the conclusion, as has been argued elsewhere (Derbinski et al. 2008; Villaseñor et al. 2008), that selection of tissue-specific genes in any given TEC is stochastic, further reflecting the unconventional mechanism of transcriptional regulation by AIRE (Mathis and Benoist 2009; Tykocinski et al. 2010). While it remains plausible that sub-populations of *Aire*-positive TEC exist (possibly reflecting distinct developmental stages), we suggest that a stochastic sampling of the silenced genome constitutes the most plausible mode of action for AIRE to contribute to efficient central tolerance induction.

The transcriptomes of *Aire-*positive and -negative mature mTEC are, except for genes regulated by *Aire* expression, remarkably similar (Supplemental Figs. 4A, 8D). This surprising result suggests that these populations constitute largely identical cell types differing only in the consequences of *Aire* expression. Given this similarity and the pervasive presence of RANK ligand, CD40 ligand, and lymphotoxin in the medulla as signals that control *Aire* expression (Anderson et al. 2013), it is surprising that *Aire* is only detected in a fraction of all mature mTEC. Although a model of sequential AIRE acquisition by mature mTEC could account for our observations, a precursor–product relationship between *Aire*-negative mature mTEC and *Aire*-positive post-mitotic mTEC has not yet been demonstrated and appears unlikely from our preliminary data. Moreover, our flow cytometric studies in *Aire*^*GFP/+*^ mice detect GFP expression in a small subpopulation of immature mTEC, suggesting that the potential for *Aire* expression is already acquired at this earlier stage of development. The molecular mechanisms that determine the acquisition and heterogeneity of *Aire* expression in immature mTEC thus remain unknown.

Two separate models have been proposed to explain the actions of AIRE. The terminal differentiation model suggests that AIRE acts in mature, terminally differentiated mTEC as a transcriptional activator that conveys the specialized property of expressing peripheral tissue-antigens (Derbinski et al. 2005). The second—the so-called developmental model—proposes that committed mTEC precursors are multipotent and use several transcriptional programs in parallel to express a broad array of tissue-specific antigens. Concomitant with their differentiation and expression of *Aire*, some of these transcriptional networks are progressively silenced so that each of the mature cells expresses either a peripheral lineage program or an undefined terminal mTEC program consequent to a loss of expression of peripheral tissue regulators (Gillard and Farr 2005). Our observations that immature mTEC express some 2620 fewer *Aire*-independent genes than mature mTEC, together with our inability to detect nonrandom transcription of genes regulated by *Aire* expression at the single-cell level, provide strong evidence in support of the first, i.e., the terminal differentiation model.

The recognition of H3K4me0 is unlikely to constitute the sole and essential mechanism by which AIRE and its binding partners target TRAs (Zumer et al. 2013), not least because AIRE co-opts the repressive ATF7IP–MBD1 complex for the induction of immunotolerance (Waterfield et al. 2014). Our findings suggest that the TSS of many genes regulated by *Aire* expression in mature mTEC is characterized by the presence of H3K27me3 together with absent or low levels of H3K4me3. This epigenetic signature implies that AIRE is also able to screen genes that are repressed via the methyltransferase activity of the Polycomb Repressive Complex 2 (PRC2). Indeed, Polycomb complexes regulate developmental genes in multiple cell types and genomic contexts and are essential for cell fate transitions and lineage-specific gene silencing (Aloia et al. 2013). Given the strong association observed between regulation by *Aire* expression and H3K27me3 marks (Fig. 6), it is not surprising that we found that genes up-regulated by *Aire* expression are also significantly enriched for many GO terms related to system development (data not shown).

AIRE fails to directly recognize H3K27me3 (Chakravarty et al. 2009; Org et al. 2009) but may do so indirectly through its known interactions with chromatin-associated proteins (Abramson et al. 2010). AIRE has been shown to interact with Chromodomain-Helicase-DNA (CHD) members 4 and 6 (Gaetani et al. 2012; Yang et al. 2013). CHD4 binds to unmethylated H3K4 and trimethylated H3K9 (Musselman et al. 2009; Mansfield et al. 2011), whereas CHD6 is known to interact with H3K27me3 (Alfonso et al. 2011). Given these interactions, it is conceivable that AIRE identifies the repressive chromatin marks indirectly via its association with CHD molecules and subsequently sanctions transcription by overriding a repressive Polycomb chromatin state (Supplemental Fig. 13). The recognition by AIRE of chromatin state(s) rather than particular DNA sequences would explain how it positively and likely stochastically regulates the expression of so many genes in a single-cell type, and may help to understand *Aire*’s emerging immune system-independent roles in embryonic stem cells and in the testis (Schaller et al. 2008; Brahmaraju et al. 2011; Bin et al. 2012).

Finally, the unprecedented finding of a high degree of PGE in all TEC subpopulations independent of their anatomical localization or degree of maturation reflects a general characteristic of these cells that has not been described for any other cell type. This faculty is likely conferred by actions of yet unknown mechanisms that function together with AIRE to provide systematic relief from epigenetic silencing in mature mTEC.

## Methods

### Mice

C57BL/6 mice were obtained from Janvier (St Berthevin, France). A novel mouse line, designated *Aire*^*GFP/+*^, was generated to target the enhanced green fluorescent protein (EGFP) expression to *Aire*-positive cells and to abolish functional *Aire* expression in mice homozygous for this knock-in (Fig. 1A; detailed in Supplemental Methods; G Nusspaumer, S Bornschein, N Shikama, T Barthlott, S Zuklys, W Krenger, K Hafen, S Naus, J Lopez-Rios, R Zeller, et al., in prep.).

### Isolation, sorting, and immunostaining of thymic epithelial cells

Thymic stromal cells were isolated from multiple thymi and sorted according to their cell surface phenotypes (Supplemental Table 1; Supplemental Methods; Zuklys et al. 2012).

### RNA and ChIP sequencing of thymic epithelial cell populations

Biologically replicate (*n* = 2) poly(A)^+^ selected RNA-seq libraries were generated from 1 µg of total RNA (see Supplemental Methods), and 36-bp single-end sequencing was performed using an Illumina GAII Analyzer. Chromatin immunoprecipitation (ChIP) was performed as previously described (Adli and Bernstein 2011) with minor modifications and antibodies as detailed in the Supplemental Methods. Biologically replicate (*n* = 2) ChIP-seq libraries were prepared with TruSeq ChIP Sample Preparation Kit (Illumina), and 50-bp paired-end reads were sequenced on an Illumina HiSeq.

### Single-cell transcriptome sequencing

The single-cell transcriptomes of FACS sorted mature mTEC were assayed using the Fluidigm C1 platform and SMART-seq (Ramsköld et al. 2012) with spike in controls from the External RNA Controls Consortium (ERCC; External RNA Controls Consortium 2005). Per-cell libraries were generated using the Illumina Nextera XT kit and 100-bp paired-end reads generated using an Illumina HiSeq 2500. For further details, please see the Supplemental Methods.

### RNA-seq and local FDR analysis of gene expression in thymic epithelial cell populations

After genomic read alignment with TopHat (Trapnell et al. 2009) (details in Supplemental Methods), upper-quartile normalized FPKM values were quantitated either (i) with Cuffdiff (Trapnell et al. 2010) denoted “Cuffdiff FPKM,” or (ii) directly from read counts, denoted “FPKM,” with multimapping reads being assigned a fractional count value according to their number of alignment locations (for comparison see Supplemental Fig. 3B; Sims et al. 2014). As described in full in the Supplemental Methods, per-gene local FDRs (Efron 2005) were estimated using FPKM values quantitated on gene models and on a matched null set of gene models shifted into selected intergenic space. For this analysis we used the Qvality algorithm (Käll et al. 2009) to estimate sample-specific mixing proportions. Differential expression analyses were performed using the DESeq algorithm (Anders and Huber 2010) as described in the Supplemental Methods along with the details of the clustering and GO analyses.

### Identification of tissue-restricted genes

Tissue-restricted genes (Figs. 2D, 3D, 5A–E, 6A–D, 7A,C; Supplemental Table 2) were identified as described above and in the Supplemental Methods. Separately, we identified nonoverlapping sets of genes showing restricted expression in all leafs and nodes of the GNF GeneAtlas sample clustering (Supplemental Fig. 6A) using the same dynamic step criteria (Figs. 3E, 4A,B).

### Mouse ENCODE analysis

The chromatin state of Ensembl protein-coding genes in somatic tissues was assessed using ChIP-seq data from the Mouse ENCODE Project (The Mouse ENCODE Consortium et al. 2012). For selected tissues and cell types, genes overlapping Mouse ENCODE ChIP-seq peak calls were identified by intersecting Mouse ENCODE peaks with 1-kb windows surrounding TSS.

### ChIP-seq analysis

Genomic alignment with BWA (Li and Durbin 2009) yielded a minimum of 33 million uniquely mapping read pairs that were de-duplicated with Picard (http://picard.sourceforge.net) before the calculation of enrichment over input as described in the Supplemental Methods. Figures show the average enrichments of the two biological replicate experiments.

### Single-cell RNA-seq analysis

Following read alignment with the GSNAP (Wu and Nacu 2010) algorithm, per-cell gene expression copy numbers were calculated from Cufflinks FPKMs using normalization curves constructed from the ERCC spike-in controls as detailed in the Supplemental Methods. For each cell we saw a median of 3.46 million reads aligning to genomic and 0.93 million aligning to the spike-in sequences. Single-cell expression level was compared to that observed in the population (Fig. 7D) after first using a first-order polynomial linear model fitted to the population level data to normalize the single-cell expression mean values across all cells such that the genes showed an average expression ratio of 1 between the mature mTEC population and the 174 single cells.

## Data access

RNA-seq and ChIP-seq data have been submitted to the NCBI Gene Expression Omnibus (GEO; http://www.ncbi.nlm.nih.gov/geo/) under the SuperSeries accession number GSE53111.

## Supplementary Material

Supplemental Material
