# Supplementary material for: Population and single-cell genomics reveal the Aire dependency, relief from Polycomb silencing, and distribution of self-antigen expression in thymic epithelia
Source: Genome Res. 2014 Dec;24(12):1918–31. doi: 10.1101/gr.171645.113 (PMC4248310; doi:10.1101/gr.171645.113)
Supplement: Supplemental Material [file supp_24_12_1918__index.html]

Population and single-cell genomics reveal the Aire dependency, relief from Polycomb silencing, and distribution of self-antigen expression in thymic epithelia — Population and single-cell genomics reveal the Aire dependency, relief from Polycomb silencing, and distribution of self-antigen expression in thymic epithelia — Supplemental Material 

# Population and single-cell genomics reveal the *Aire* dependency, relief from Polycomb silencing, and distribution of self-antigen expression in thymic epithelia

## Supplemental Material

**Files in this Data Supplement:**

- Supplemental Material.pdf
- Supplemental Table2.xlsx
- Supplemental Table3.xlsx
- Supplemental Table4.xlsx
- Supplemental Table5.xlsx
