# Supplementary material for: Population and single-cell genomics reveal the Aire dependency, relief from Polycomb silencing, and distribution of self-antigen expression in thymic epithelia
Source: Genome Res. 2014 Dec;24(12):1918–31. doi: 10.1101/gr.171645.113 (PMC4248310; doi:10.1101/gr.171645.113)
Supplement: Supplemental Material [file supp_gr.171645.113_Supplemental_Material.pdf]

Supplementary Material

Contents

I. Supplementary Methods

II. Supplementary Figure Legends

III. Supplementary Figures

IV. Supplementary Table Legends

V. Supplementary Tables

VI. Supplementary References

Supplementary Methods

Mice

In brief, $Aire^{GFP/+}$ mice were generated using a previously described vector (Ivanova et al. 2005) that was adapted as a knock-in targeting vector to replace the first exon of the *Aire* locus with the sequence of EGFP. Following 129/Ola embryonic stem cell (ESC) electroporation and screening for homologous recombinants by Southern blotting and PCR, verified ESC were used for blastocyst injections. Germ-line transmitted heterozygous mice were bred onto a C57BL/6 genetic background. Heterozygous and homozygous mice were born at the expected Mendelian frequency. The transgenic mice displayed a thymic cellularity of lymphoid and stromal cells that was indistinguishable from that of wild type mice (data not shown). Mice were maintained under specific pathogen-free conditions and according to Swiss federal and institutional regulations.

Isolation and sorting of thymic epithelial cells

Fragmented thymi were digested repeatedly for 15-20 min at 37 °C with 1mg/ml Collagenase/Dispase (Roche Diagnostic) and 100μg/ml DNaseI (Roche Diagnostic) in HBSS containing 2% FCS (Perbio), to obtain single cell suspensions. After the final digest, cells were pooled and labelled with biotinylated anti-EpCAM for positive enrichment by AutoMACS system (Miltenyi Biotec), and stained using the following directly labelled antibodies and reagents: FITC-anti-IAb (clone AF6-120.1, BioLegend), PE-anti-Ly51 (clone 6C3, BioLegend), PECy7-anti-CD45 (clone 30-F11, BioLegend), APC-Cy7-anti-I-A/E (clone M5/114.15.2, BioLegend), biotinylated anti-EpCAM (clone G8.8, DSHB, University of Iowa), Streptavidin-labelled PerCP-Cy5.5 (BioLegend) and Cy5-UEA1 (Vector Laboratories). The cells were exposed to 4', 6-diamidino-2-phenylindole (DAPI) to identify dead cells and then sorted by flow cytometry (FACSAira, BD Biosciences) achieving a TEC purity of over 93%. Sorted TEC were pelleted, lysed in the RLT lysis buffer of the RNeasy Micro Kit (Qiagen), or cross-linked for ChIP and kept at -80 °C until use.

Immunohistochemistry

Freshly isolated thymus tissue was fixed in PBS containing 1% PFA and 30% sucrose for 1 hour at 4°C, and then embedded in OCT (Cell Path) for 7 μm sections. The sections were subjected to heat-induced antigen retrieval using 10 mM Tris HCl (pH9) containing 1mM EDTA buffer, prior to antibody staining using FITC-anti-GFP (ab6662, Abcam), anti-AIRE (clone 5C11-4, kindly provided by H. Scott, Adelaide, or M-300 (sc-33189, Santa Cruz)), Alexa555-anti-rat IgG (A21434, Invitrogen) and Alexa555-anti-rabbit IgG (A21429, Invitrogen). Images were acquired using a Zeiss LSM510 and its software.

RNA-seq of thymic epithelial cell populations

Total RNA was recovered from TEC isolated from multiple thymi using the RNeasy Micro Kit. Concentration, quality, and integrity were assessed by NanoDrop 1000 (ThermoScientific) and BioAnalyzer (Agilent laboratories). RNA-seq libraries were prepared from 1 µg of total RNA using the mRNA-Seq Sample Prep Kit (Illumina).

ChIP-seq

Chromatin immunoprecipitation (ChIP) was performed as previously described (Adli and Bernstein 2011) with the following modifications: Protein G magnetic beads (Dynabeads, Life Technologies) were used to capture antibody-chromatin complexes. The captured complexes were washed four times with wash buffer (0.1 % SDS, 1 % Triton X-100, 0.1 % deoxycholate, 20 mM Tris- HCl (pH 8), 1 mM EDTA, 0.5 mM EGTA) containing 150 mM NaCl, once with wash buffer containing 500 mM NaCl, and finally once with LiCl wash buffer (250 mM LiCl, 1 % NP-40, 1% deoxycholate, 10 mM Tris- HCl (pH 8), 1 mM EDTA). Antibodies used were anti-H3K4me3 (ab8580, Abcam) and anti-H3K27me3 (#9733, Cell Signalling).

Single cell transcriptome sequencing

Single cells were isolated and amplified on the Fluidigm C1 platform at the Sanger Institute/EBI Single Cell Genomics Centre. Briefly, a pool of FACS sorted TECs (220K cells/ml) was loaded on two medium chips (designed for capture of cells between 10-17 µM in diameter). After loading and capture, chips were inspected under the microscope and the presence of a single cell at 191/192 capture sites was confirmed.

Cell lysis, cDNA synthesis and subsequent PCR amplification was performed using the SMARTer Ultra Low RNA Kit (Clontech) for the C1 platform as per the manufacturer's instructions (Clontech/Fluidigm). The cell lysis buffer (20 µL) was supplemented with 1 µL of a 1:400 dilution of the External RNA Controls Consortium (ERCC, (External 2005)) spike-in Mix 1 (Invitrogen).

Indexed Libraries from the 96 samples harvested from each chip were prepared using the Nextera XT kit (Illumina). Multiplexed libraries from each chip were sequenced over two lanes on a HiSeq 2500 (Illumina) at the Wellcome Trust Sanger Institute run in fast mode to generate 100bp paired end reads.

RNA-seq analysis of thymic epithelial cell populations

RNA-seq reads were mapped to the mouse genome (mm9) by providing TopHat (Trapnell et al. 2009) with a transcriptome index built from Ensembl protein coding gene models and a junctions file computed from Ensembl mRNAs. Genes refer to Ensembl version 67 protein coding genes on assembled chromosomes. For differential expression analysis the DESeq algorithm (Anders and Huber 2010) was used to test differences in the mean number of reads mapping to gene models in a biologically replicated design (n=2). Hierarchical clustering of TEC population correlation distances was performed using average linkage clustering in R. Leaf order was optimised using the R "cba" package and significant clusters identified using boot-strapping analysis as implemented in the R "pvclust" package (Suzuki and Shimodaira 2006). GO enrichments were identified using annotation from MGI and a hypergeometric test.

Identification of tissue-restricted genes

Tissue restricted genes were identified using data from the GNF Mouse GeneAtlas V3 (Lattin et al. 2008) (GEO accession GSE10246). Raw microarray expression data from the Atlas were normalised together using the R "rma" package and expression values for 64 non-thymic physiological samples were calculated (replicate values averaged). In order to avoid representation bias, GNF GeneAtlas samples were hierarchically clustered into 35 groups (Supplementary Fig. 6A). To identify tissue-restricted expression, we used a novel dynamic step method. Because microarray background levels are probe-specific, we tested each probe separately for tissue-restricted expression. Guided by thresholds typically chosen for microarray data analysis, we define tissue restricted probes as those with a minimum normalised expression value of 50 that showed a moderated exponential step-up in expression, such that expression was substantially higher in 1-5 tissue groups than in the 6th highest tissue group (Supplementary Fig. 6B and 6C). Genes with unanimously tissue-restricted probe sets were designated as tissue-restricted (Fig. 2D, 3D, 5A-E, 6A-D, 7A and C, and Supplementary Table 2).

Calculation of per gene Local FDR

Per gene local FDRs were determined using FPKM values calculated directed from read counts (multi-mapping reads were assigned a fractional count value according to their number of alignment locations) because probabilistic methods such as Cuffdiff cannot provide expression estimates for lowly expressed genes. To calculate the null (background) distribution of FPKM values, collapsed gene models (introns shrunk to 50bp) were shifted into proximal, mappability-matched intergenic (>5kb from known Ensembl transcripts) space devoid of ESTs (UCSC annotation) (Supplementary Fig. 3A). FPKM values were then quantitated on protein coding gene models and the matched background set (Supplementary Fig. 3B). Local FDRs (Efron 2005)

(Supplementary Fig. 3E) were determined using sample specific mixing proportions estimated using the Qvality algorithm (Käll et al. 2009).

Single-cell RNA-seq analysis

Reads were aligned using the GSNAP (Wu and Nacu 2010) algorithm with the parameters " -N1 --maxsearch=1000 -w 500000 --pairexpect=100 --pairdev=200 -n100" to a custom genome based on the mm10 version of the mouse genome and containing the ERCC spike-in sequences. Gene expression was quantitated separately for each cell using Cufflinks against Ensembl 74 reference annotations with the parameters " --multi-read-correct --max-intron-length 500000 --library-type=fr-unstranded --frag-bias-correct --no-effective-length-correction --max-bundle-length 10000000 --max-bundle-frags 2000000 --min-intron-length 50 --max-mle-iterations 10000". Copy numbers were then calculated for protein coding genes based on per-cell normalisation curves determined from known spike-in copy number and observed spike-in FPKMs. The curves were obtained from a first order polynomial linear model forced through the plot origin (good linearity observed, see Supplementary Fig. 11A).

ChIP-seq analysis

Reads were aligned to the mouse genome (mm9) using BWA (Langmead et al. 2009) ("bwa aln -l 25 -k 2 -n 0.1 -q 20"). Following de-duplication using Picard, ChIP enrichment over input was calculated using either (i) MACS2 ("bdgcmp -m FE") after preparing normalised bedgraphs for ChIP and input samples using MACS2 (Zhang et al. 2008) ("callpeak --SPMR") (Fig. 6A,C) or by (ii) calculating normalized read counts for 1kb TSS windows computing the ratio of ChIP/input for

these windows (Fig. 6B,D-F). Meta-gene profile plots (Fig. 6A & C) were prepared using CEAS (Shin et al. 2009) and data visualised using the UCSC Genome Browser (Kent et al. 2002).

Supplementary Figure Legends

Supplementary Figure 1: Sorting strategy for TEC subpopulations. Sorting strategy for (A) mTEC $MHCII^{hi}$ and mTEC $MHCII^{lo}$ from 4 week-old C57BL/6 mice, and (B) their sort purity. (C) Sorting strategy for GFP^{+} and GFP^{-} mTEC $MHCII^{hi}$ subpopulations from 4 week-old *Aire:GFP* mice and (D) their sort purity. (E) Sorting strategy for cTEC and mTEC subpopulations from 1 week-old C57BL/6 mice and (F) their sort purity. Live (i.e. DAPI-negative) TEC ($CD45^{-}$ $EpCAM^{+}$), were differentiated into cTEC and mTEC using the expression of Ly51 and reactivity with the UEA-1 lectin, respectively.

Supplementary Figure 2: $Aire^{GFP/GFP}$ mice are Aire deficient. (A) Flow cytometry analysis of Aire and GFP expression in mTEC of Aire GFP/GFP mice (top) and Aire +/+ mice (bottom). TEC were subjected to Aire intracellular staining and assessed for Aire (left) and GFP (right) expression. The numbers indicate the percentage of Aire and GFP expressing cells, respectively, in total mTEC. The number of GFP expressing cells was underrepresented since GFP signals were reduced by cell permeabilisation for intracellular staining of Aire protein. (B) Aligment of RNA-seq reads to the *Aire* locus from *Aire*$^{GFP/+}$ and *Aire*$^{GFP/GFP}$ mTEC that express GFP (*Aire*-positive and *Aire*-knockout mTEC), demonstrates transcription of the *Aire* gene to be almost completely abolished by the introduction of the stop cassette into exon 1 (Fig. 1A). Note that residual transcription remains from the 5'UTR.

Supplementary Figure 3: Local FDR for RNA-seq. Calling genes as expressed or not from RNA-seq data remains an open challenge. Previous global FDR approaches are not informative on a per-gene basis (Ramsköld et al. 2009). Here we used a novel local FDR approach to estimate per-gene false discovery rates (Supplementary Table 2). (A) A major source of background noise in RNA-seq experiments derives from genomic DNA contamination. To estimate this noise, the expression level of neighbouring intergenic sequence was quantitated by translocating the gene set of interest (red) into a proximal, mapability matched intergenic space (at least 5kb from known transcripts) devoid of UCSC ESTs to form a "background" gene set (blue; introns are shrunk to 50bp). (B) From these gene models, upper-quartile normalized FPKM values were calculated that showed a high correlation with upper-quartile normalised Cuffdiff FPKMs (Supplementary Table 2). These values were used because Cuffdiff does not report FPKM values for lowly expressed genes. (C) Foreground (red) and background (blue) FPKM distributions were determined for each TEC sample. (D) Here the foreground FPKMs were used to estimate the mixture density and the background distribution formed the null density in order to calculate local FDR scores as defined by Bradley Efron (Efron 2005). We used the non-parametric algorithm Qvality (Käll et al. 2009) to estimate the mixing proportion, P_0 . The scaled null distributions are depicted by the black lines in (C). The dashed lines and accompanying numbers in (C) indicate the FPKM threshold at which genes are detectable at <5% local FDR. (E) Application of the previously described (Ramsköld et al. 2009) global FDR approach results in the detection of a similar number of genes as being expressed in the TEC populations (see main Fig. 2A). (F) The background distribution is elevated in the mature *Aire*-expressing TEC populations (lower panels C), a pattern seen more clearly in a heatmap of background FPKM values. This may be due to higher levels of genomic contamination in these samples or alternatively may

represent the presence of un-annotated genes that are up-regulated by *Aire*, or increased run-on or intergenic transcription in the presence of *Aire*.

Supplementary Figure 4: DE-seq analysis and expression of tissue-restricted genes in the TEC populations. (A): Hierarchically clustered symmetrical heatmap showing the total number of genes differentially expressed by all pairwise combinations of TEC populations at >2 fold (5% FDR) using the DESeq algorithm(Anders and Huber 2010) (Supplementary Table 3). (B) Numbers of up-and down-regulated genes from (A) are shown. (C) Heat map of the expression of tissue-restricted genes (genes expressed in 1-5 GNF GeneAtlas tissue groups by the dynamic step criteria, see Supplementary Methods and Supplementary Fig. 6) in cTEC, *Aire*-negative and *Aire*-positive mature mTEC (see Supplementary Fig. 6). (D) Numbers of tissue-restricted genes expressed by cTEC, *Aire*-negative and *Aire*-positive mature mTEC and of those genes that are commonly expressed between these three TEC subtypes at the indicated FPKM threshold. Non-redundant numbers are depicted as indicated by the colours used in the Venn diagram. For example, the black line represents the number of tissue-restricted genes commonly expressed by cTEC, mature *Aire*-negative mTEC and *Aire*-positive mature mTEC, while the green line indicates the number of tissue restricted genes detected in cTEC. Numbers in the inset represent the number of tissue-restricted genes detected at an FPKM threshold of 0.13 (the threshold at which genes are detectable at <5% local FDR in all TEC populations).

Supplementary Figure 5: Gene ontology (GO) analyses of genes not detected in mTEC. GO analysis was performed using MGI annotation and a hypergeometric test to identify GO

categories that were significantly enriched (<5%FDR). (A) Enrichment of biological process, cellular location, and molecular function categories. (B) Clustering of all categories by dissimilarity of gene membership. Representative categories, chosen after cutting the dendrogram at the level indicated, are highlighted in red and shown in Fig. 2C. (C) Heatmaps of the expression of Olfactory and Vomeronasal receptors in the indicated TEC populations.

Supplementary Figure 6: Identification of genes with tissue-restricted expression from the GNF GeneAtlas: (A) Non-thymic physiological samples from the mouse GNF GeneAtlas were reduced to 35 groups by hierarchical clustering and pruning the dendrogram tree at the indicated level (dashed red line) in order to avoid the over-representation of similar tissue types. (B) After ranking groups by expression level, the dynamic step method calls tissue specific genes as those with expression levels in a few (*j*) groups that are exponentially higher than that in the next highest group (*n*-*j*th) group. (C) The dynamic step function sets a threshold based on a moderated exponential jump. (D) The overlap between tissue specific genes called using the dynamic step method and a previously used thresholding method (Gardner et al. 2008). The data indicate a higher sensitivity for the dynamic step method. (E) Comparison between genes called as tissue specific by only one of the two methods. Genes unique to the threshold method (green) show only weakly higher expression in 1-5 tissues whereas genes unique to the dynamic step method (blue) show a large step up in expression in 1-5 tissues. (F) Percentage of calls from the two methods that represent known housekeeping genes. While calls common to both methods or to the dynamic step method alone are markedly depleted in housekeeping genes, calls specific to the threshold only method show a greater enrichment for housekeeping genes demonstrating the improved specificity of the dynamic step method. Tissue specificity calls are provided in Supplementary Table 2.

Supplementary Figure 7: Pathway analysis of the TEC lineage. (A) Clustering of the TEC populations on non-promiscuous genes define three significant (p>0.95) clusters of TEC: cTEC, immature mTEC and mature mTEC. (B) Identification of KEGG pathways enriched >2 fold (hypergeometric test, <5% FDR) in genes differentially expressed between the three major TEC types (DESeq analysis, 5% FDR, Supplementary Table 3 & Supplementary Table 4). Numbers in white indicate the number of pathway genes differentially expressed in each comparison. Black squares represent the absence of significant enrichment. The expression of genes in the two underlined pathways is shown in full in panels C and D: Expression of the Jak-STAT signalling pathway (C), and NF-kappa B signalling pathway genes (D) in the three major TEC types. Rows are hierarchically clustered.

Supplementary Figure 8: TEC marker analysis. (A-C) Identification of genes differentially expressed between the three major TEC types. (D) Mature mTEC positive for *Aire* expression but lacking functional *Aire* (mature *Aire*-knockout mTEC) have virtually identical transcriptional profiles to mature *Aire*-negative mTEC. (E) Genes characteristic of a TEC type were identified as those highly expressed (>10 FPKM) at a level at least twice that observed in the TEC type with the next highest expression level. (F) Heatmap showing the expression of the top 20 candidate markers for each of the three types of TEC. For the full list of these genes see Supplementary Table 5.

Supplementary Figure 9: Gene ontology analysis of H3K27me3 marked genes in mature mTEC: Gene ontology analysis of genes whose TSS's (1kb centered window) showed an average (n=2 ChIP-seq replicates) 2 fold or greater enrichment for H3K27me3 relative to input in mature mTEC. A representative selection of gene categories is shown. Analysis performed using

GOToolBox (http://genome.crg.es/GOToolBox/) (Martin et al. 2004), Benjamini-Hochberg corrected p-values from a Hypergeometric Test are shown.

Supplementary Figure 10: Examples of the chromatin state of genes up-regulated by *Aire:* The H3K4me3 and H3K27me3 status of six genes up-regulated by *Aire* in mature mTEC. Plots were generated using the UCSC Genome Browser (https://genome.ucsc.edu/).

Supplementary Figure 11: Single cell RNA-seq normalisation curves and sensitivity analysis: (A) The spike-in normalisation curves for nine randomly selected cells are depicted. A first order polynomial model (forced through the origin) was fitted to log 10 (x+1) transformed copy number and FPKM values of the ERCC spike-in controls (red curve) and used to predict the copy number of protein coding genes. (B) Detection sensitivity for the ERCC spike-in controls indicates a robust detection of molecules with a copy number of 20 (dashed line) or above. (C) Aggregate numbers of genes detected across the single cells at the given copy number thresholds. The median number of genes expressed for a given number of single cells was calculated by repeated random sampling.

Supplementary Figure 12: Single cell analysis of 174 mature thymic epithelial cells: Mature (4 week old) thymic epithelial cells were sorted and subjected to transcriptomic sequencing as described in Supplementary Methods. (A) The mean expression of genes across all 174 mTEC correlates well with their expression level separately determined from the mature mTEC population. (B) The distribution of *Aire* expression levels among single mature mTEC. (C) We found a significant correlation (Spearmans r: 0.40 p = 4.24×10^{-8}) between the number of genes detected in a cell and the expression level of *Aire.* (D) We compared the fraction of cells expressing the given TRA that we observed with that previously reported using a PCR approach (Derbinski et al. 2008; Villaseñor et al. 2008), finding good agreement. (E) The histogram shows

the distribution of the fraction of single mTEC in which a gene is expressed for the given non-overlapping gene sets.

Supplementary Figure 13: A model for the recognition of Polycomb silenced genes by AIRE. While AIRE is not thought to recognize H3K27me3 directly (Koh et al. 2008; Org et al. 2008), we propose that in addition to recognising unmethylated H3K4 via its PHD1 domain, AIRE may be recruited to sites of H3K27 trimethylation via CHD interaction partners, the most likely of which being CHD6 due to its demonstrated interaction with H3K27me3 (Gaetani et al. 2012; Yang et al. 2013). Upon recruitment to these locations, AIRE may override the repressive histone mark (left panel), or may act by locally reprogramming chromatin to a state permissive of transcription (right panel). If AIRE acts to override a repressive state we would predict this state to identify AIRE-targeted genes whether they are actively transcribed or silent in an individual cell. In contrast, should AIRE binding result in a reprogramming of the local chromatin state as a prerequisite for PGE, we anticipate the chromatin state of AIRE target loci to be heterogeneous at the population level. Both models are compatible with the notion that AIRE and its interaction partners target inactive promoters which have already bound the basal transcriptional machinery but where the RNA polymerase II is either stalled or produces only sterile and unstable transcripts (Sims et al. 2004; Oven et al. 2007; Abramson et al. 2010). DNA-PK: DNA-dependent protein kinase; H3: Histone 3, K: Lysine; TOP2A: DNA topoisomerase 2-alpha; POL II: RNA polymerase II; P-TEFb: positive transcription elongation factor b, CHD: Chromodomain-helicase DNA-binding protein.

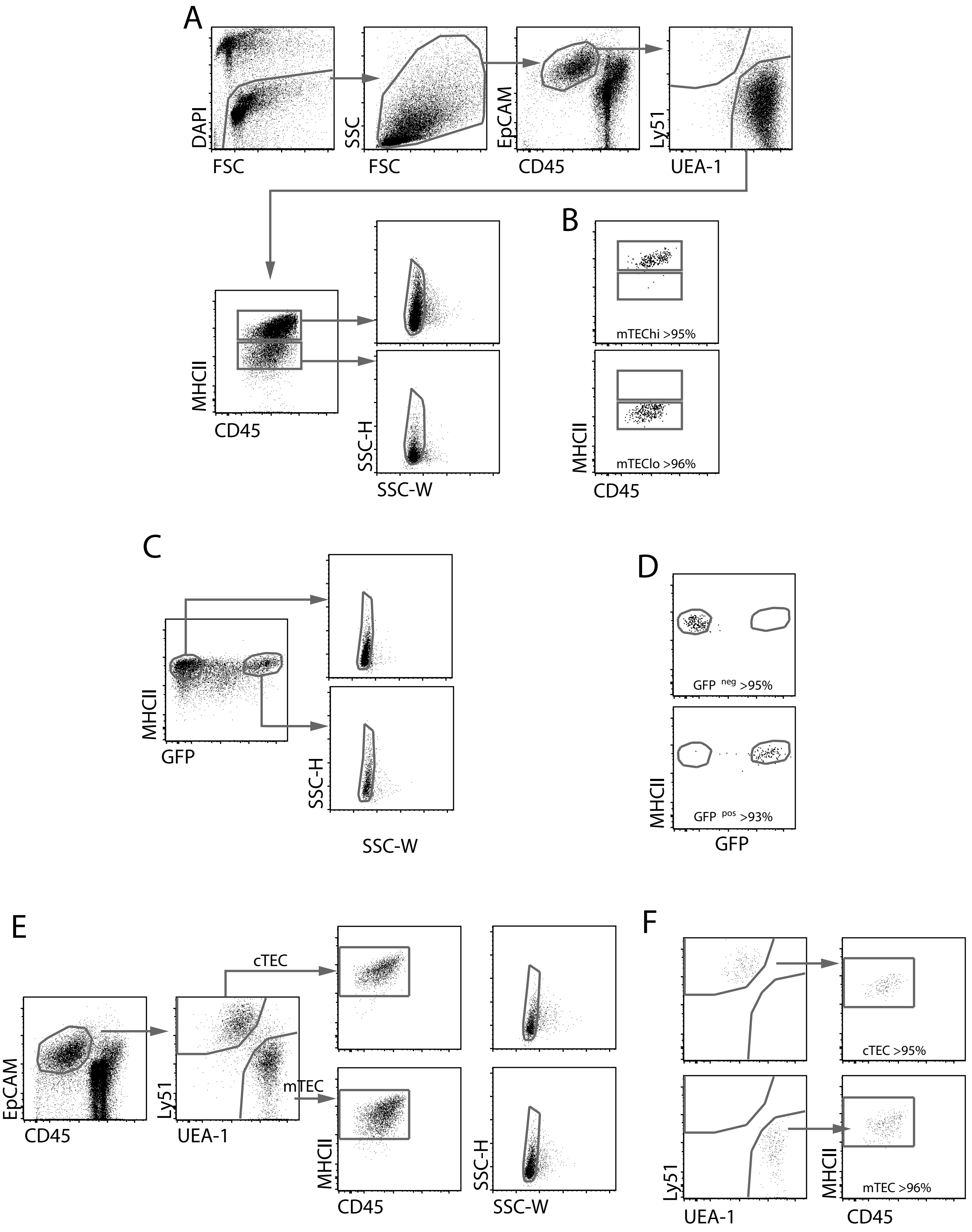

Supplementary Figure 1

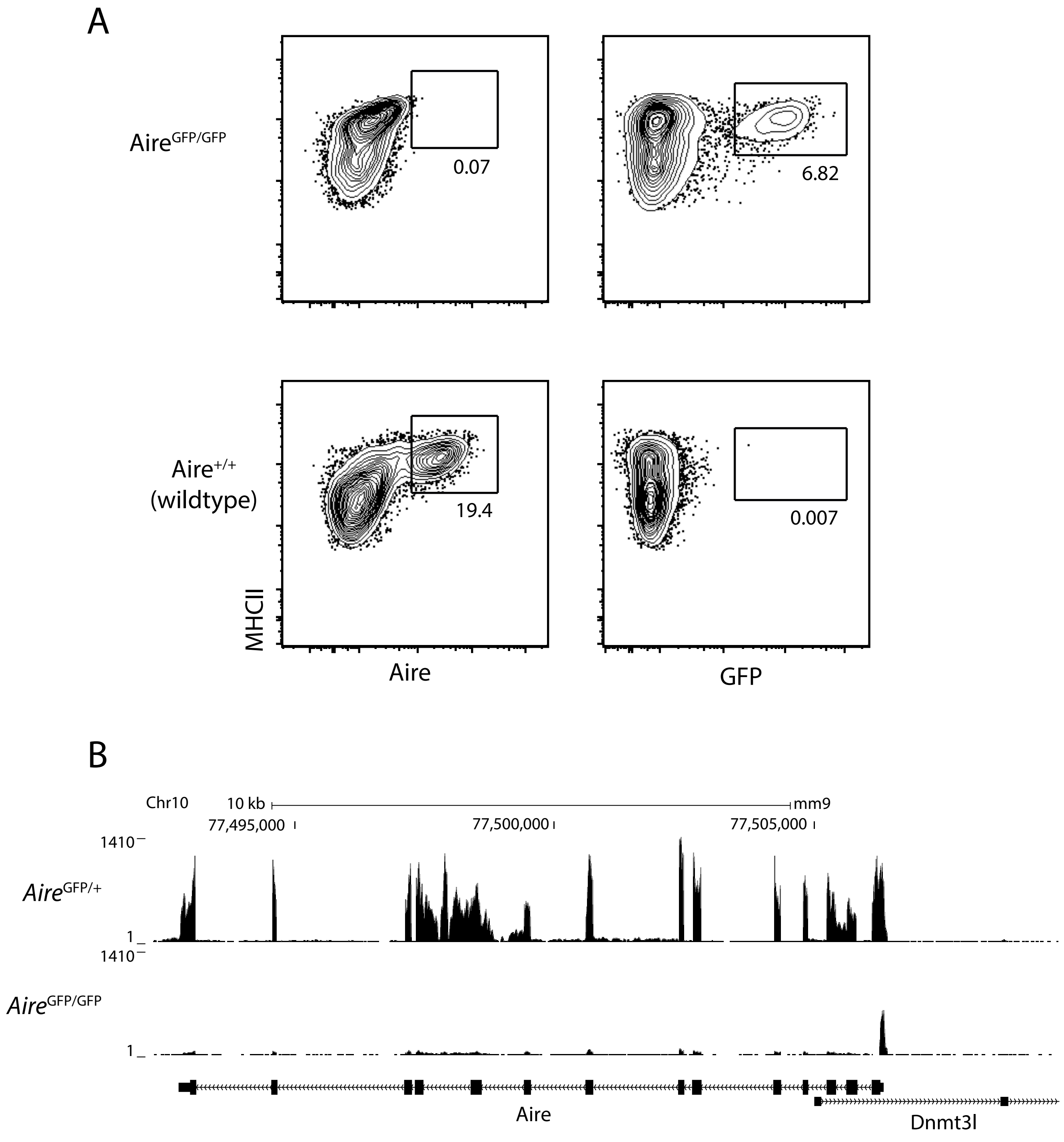

Supplementary Figure 2

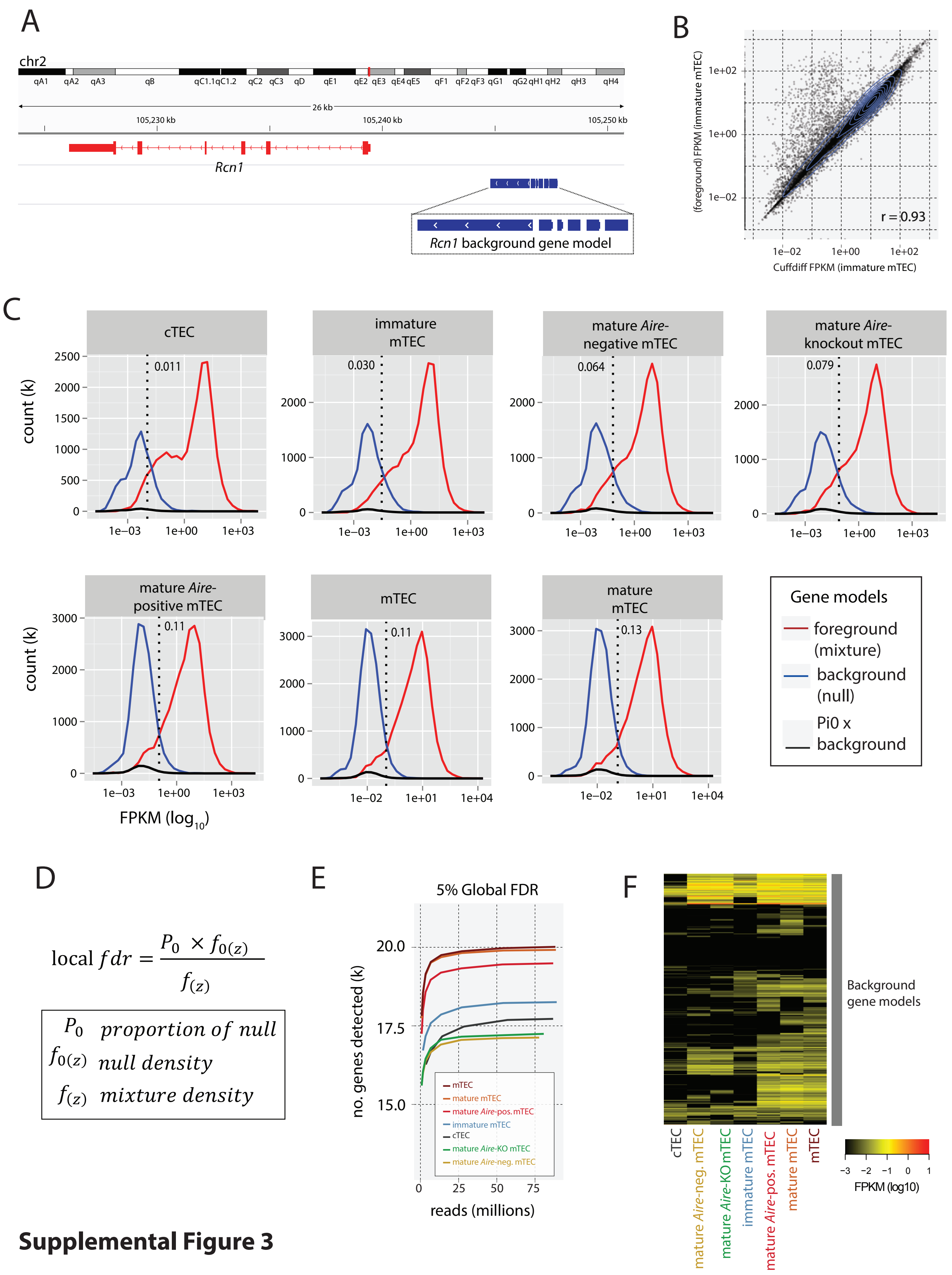

Supplemental Figure 3

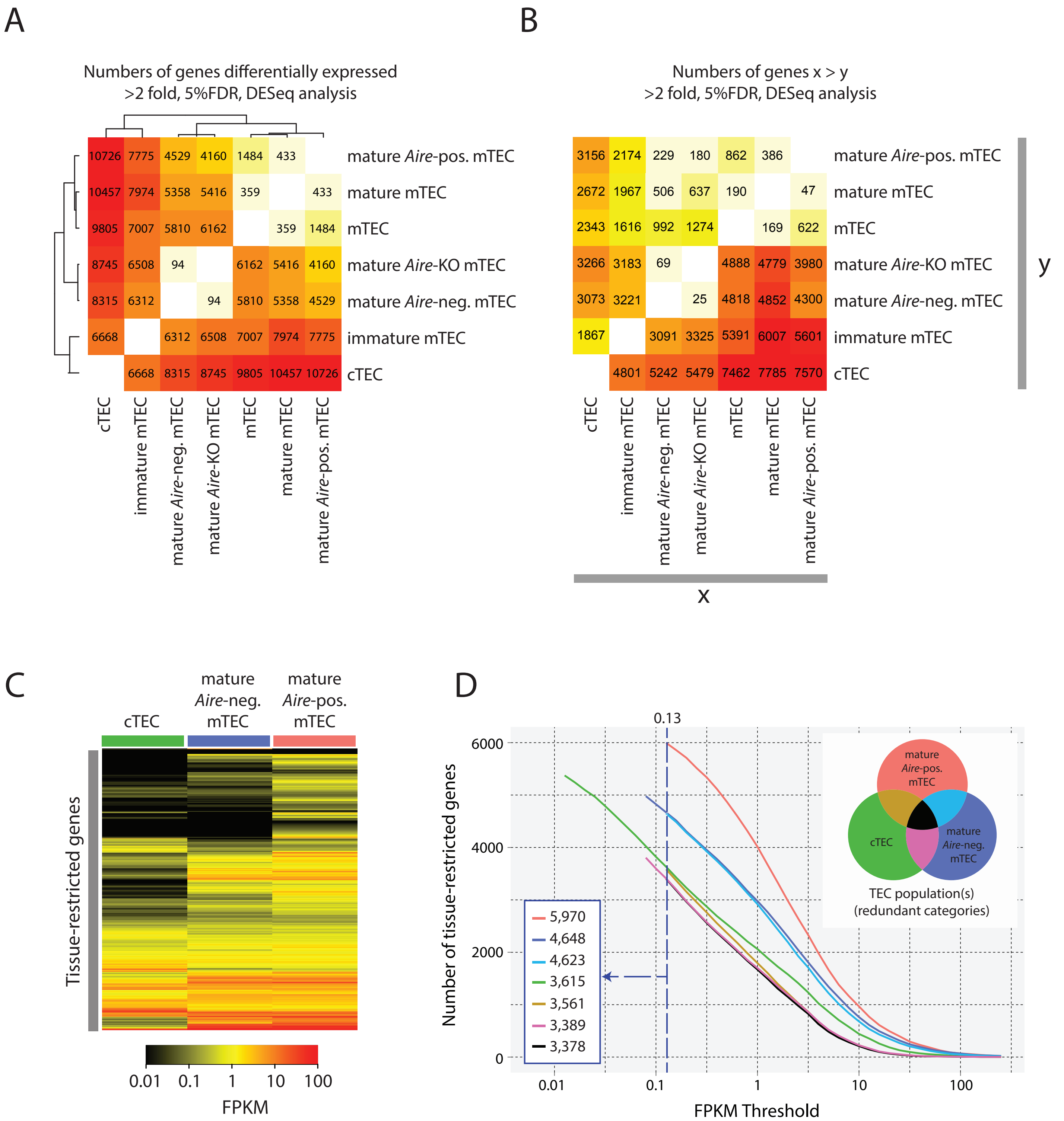

Supplemental Figure 4

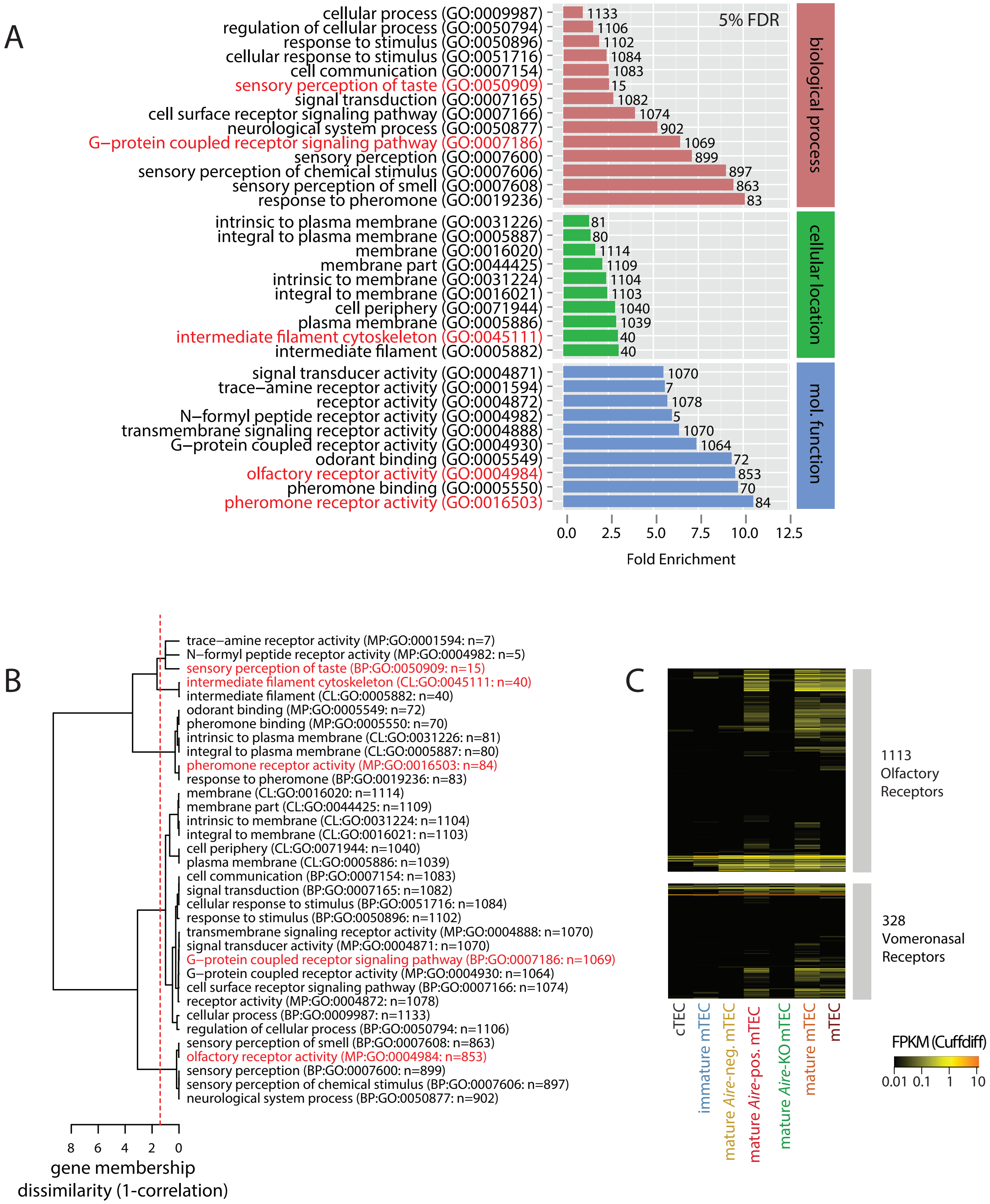

Supplemental Figure 5

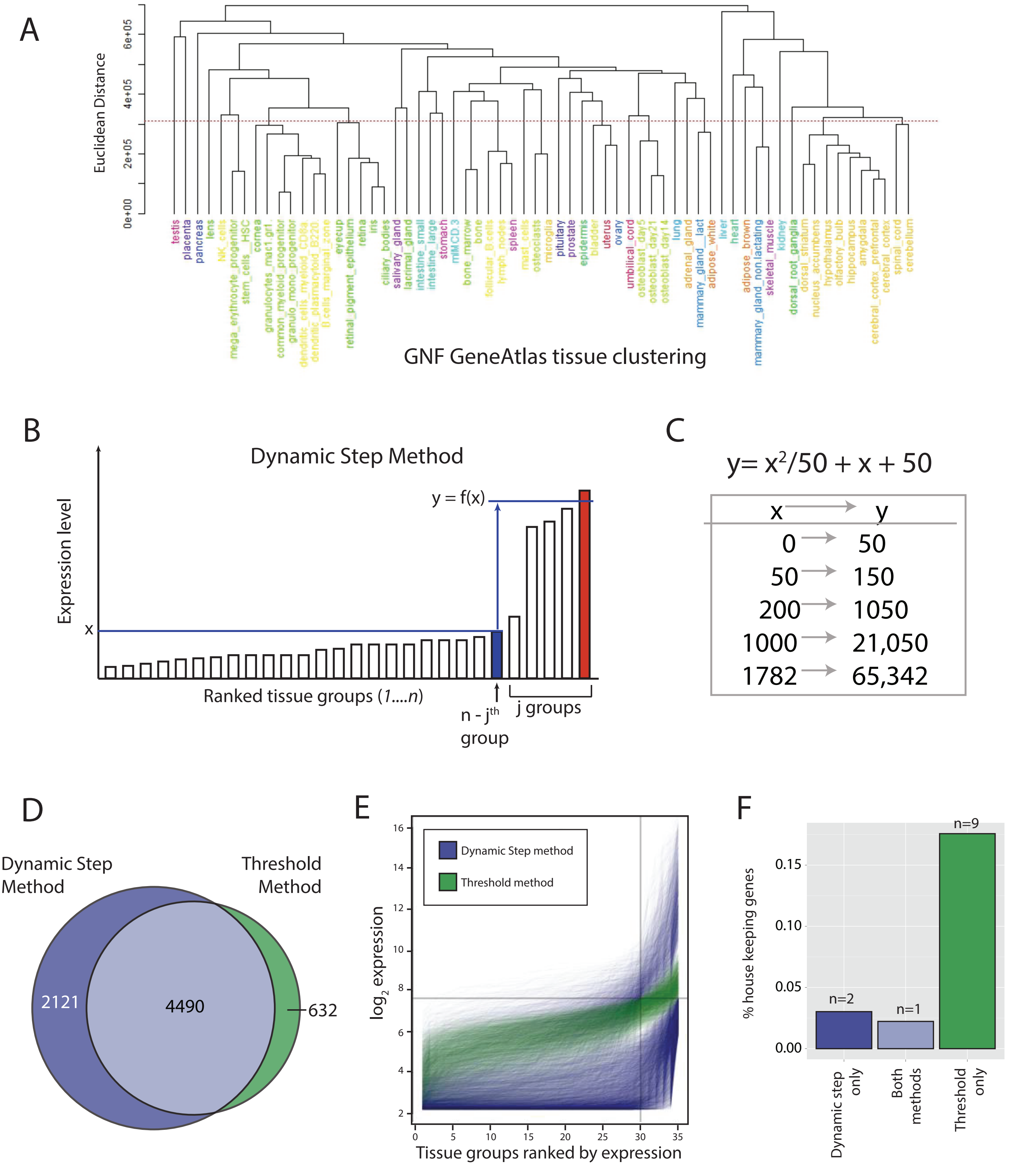

Supplementary Figure 6

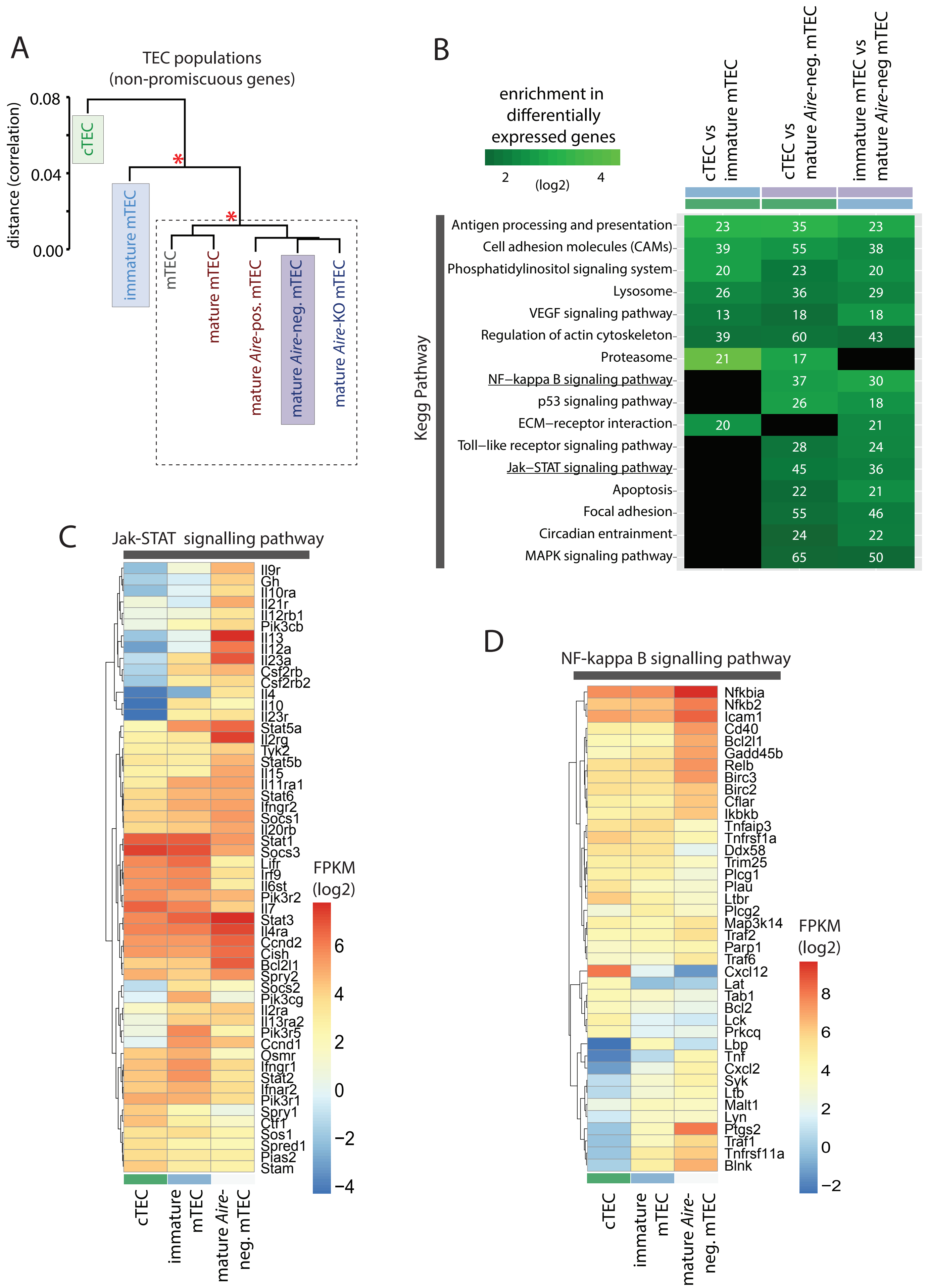

Supplemental Figure 7

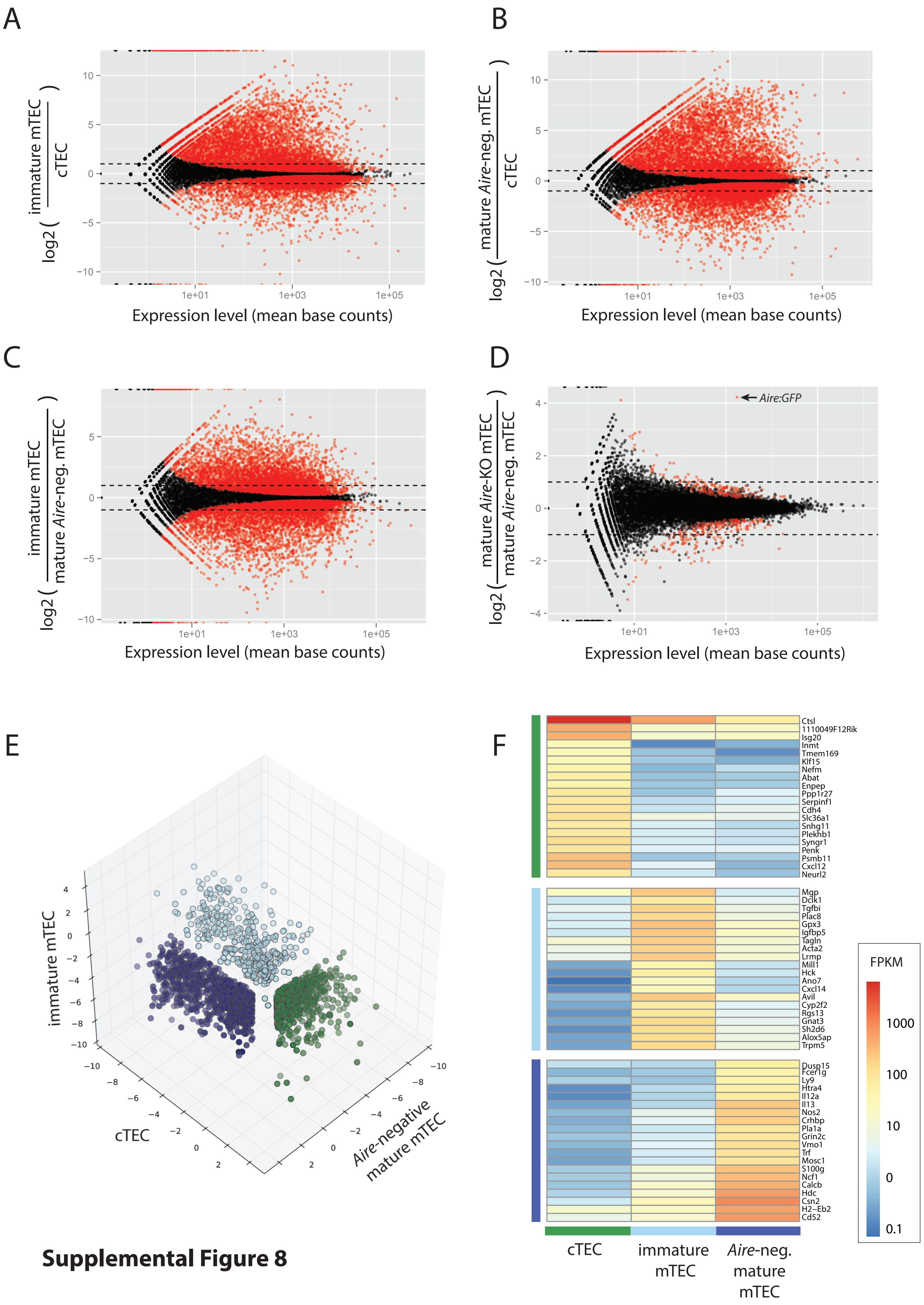

Supplemental Figure 8

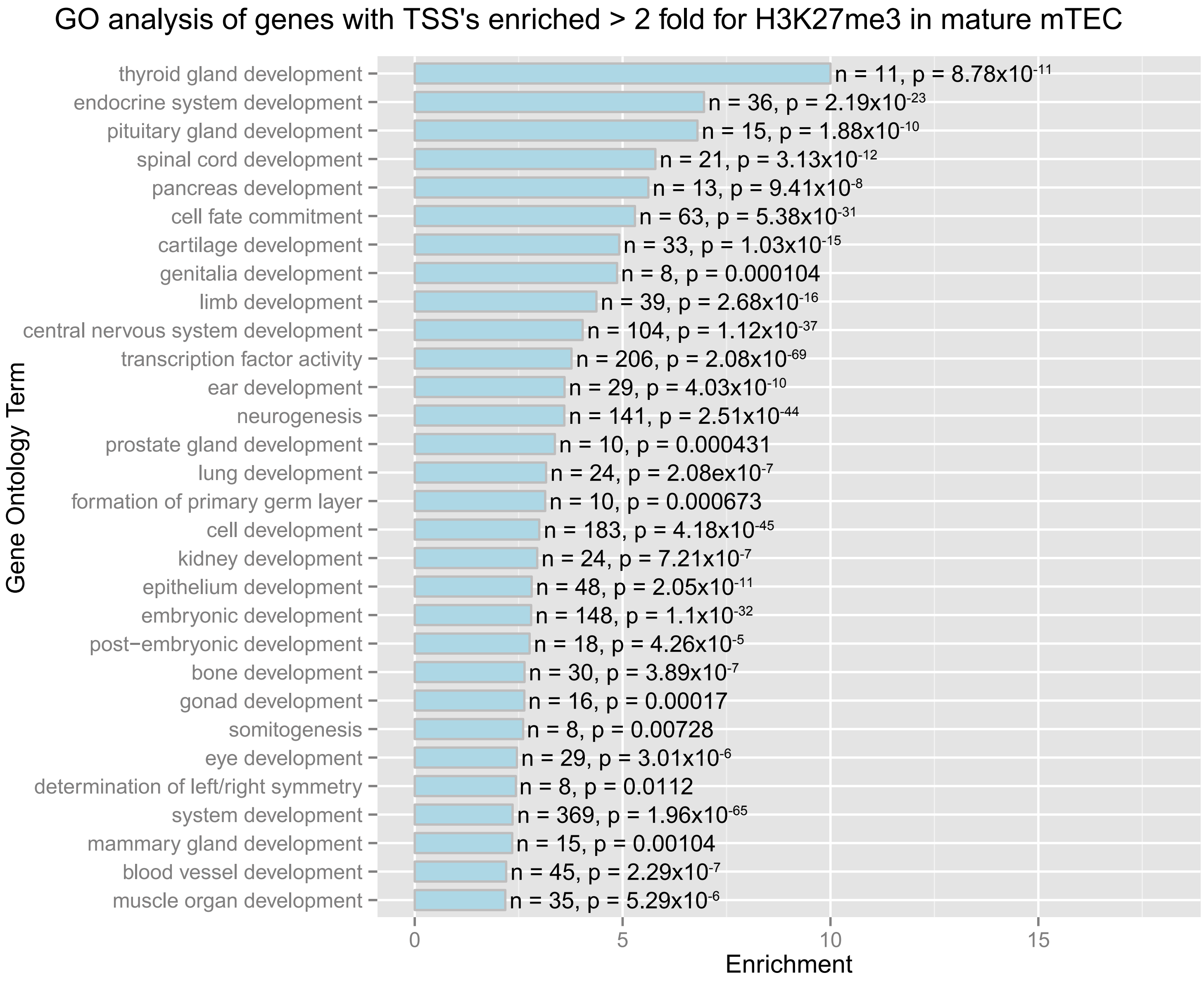

Supplementary Figure 9

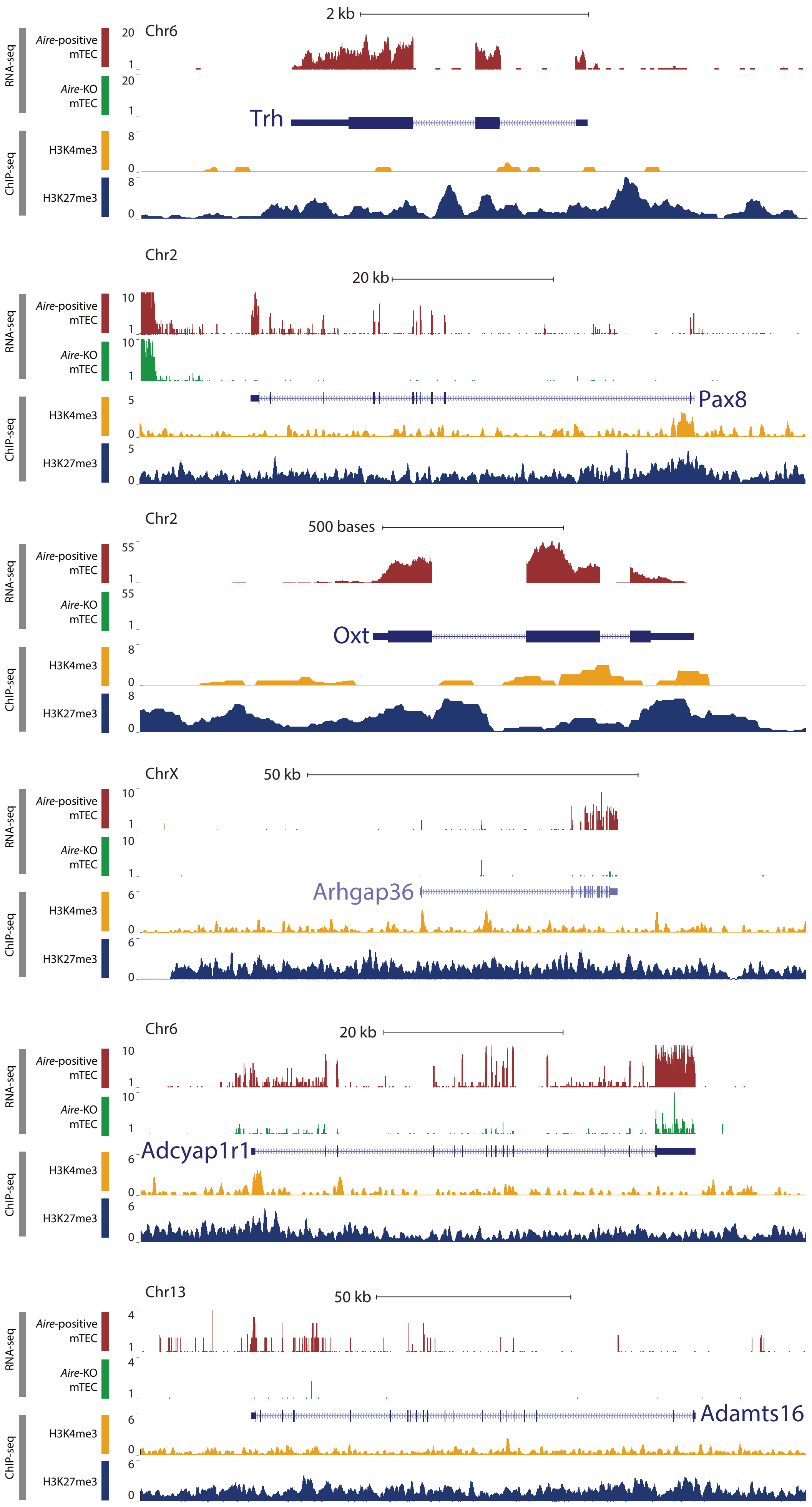

Supplementary Figure 10

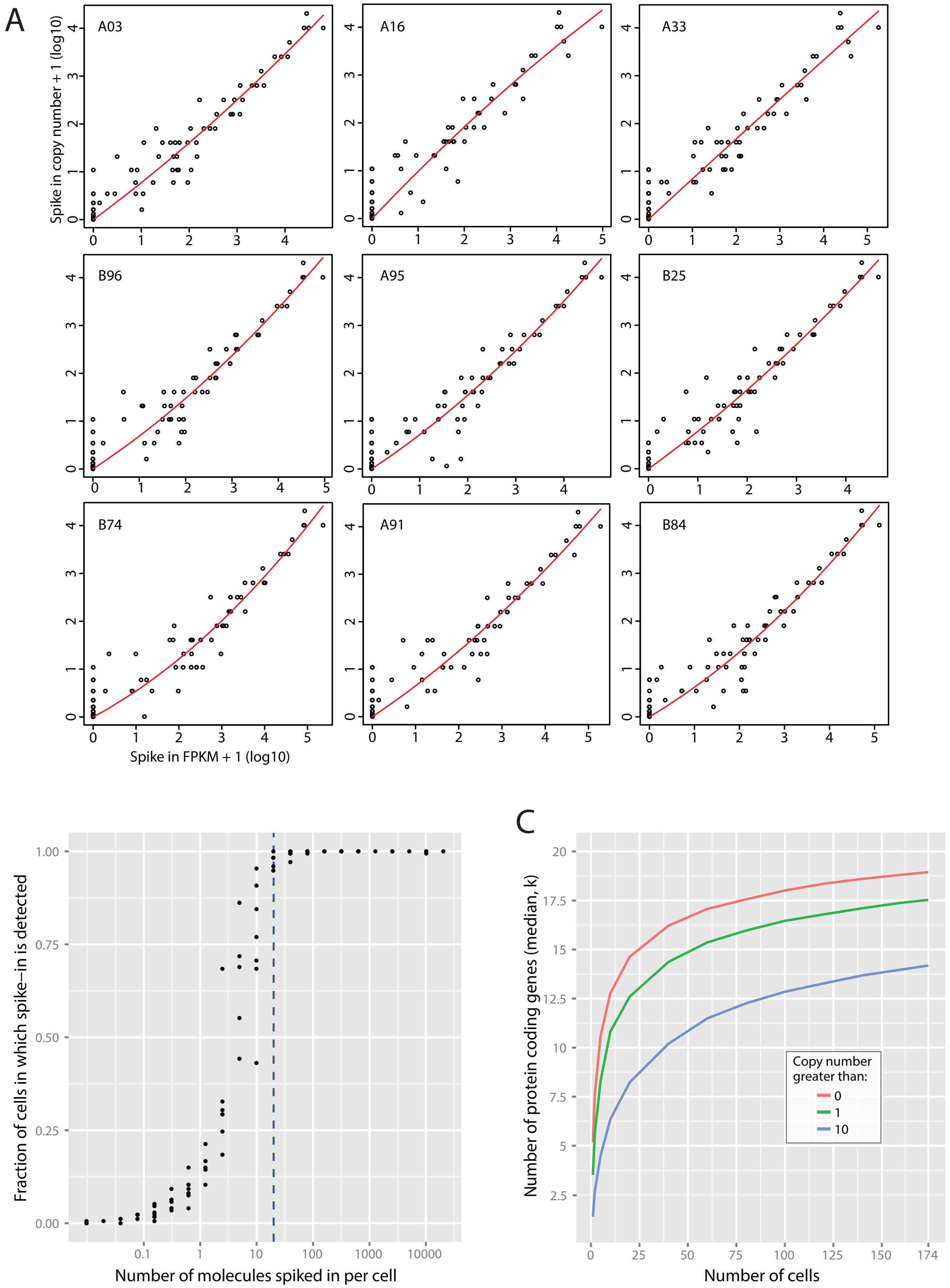

Supplementary Figure 11

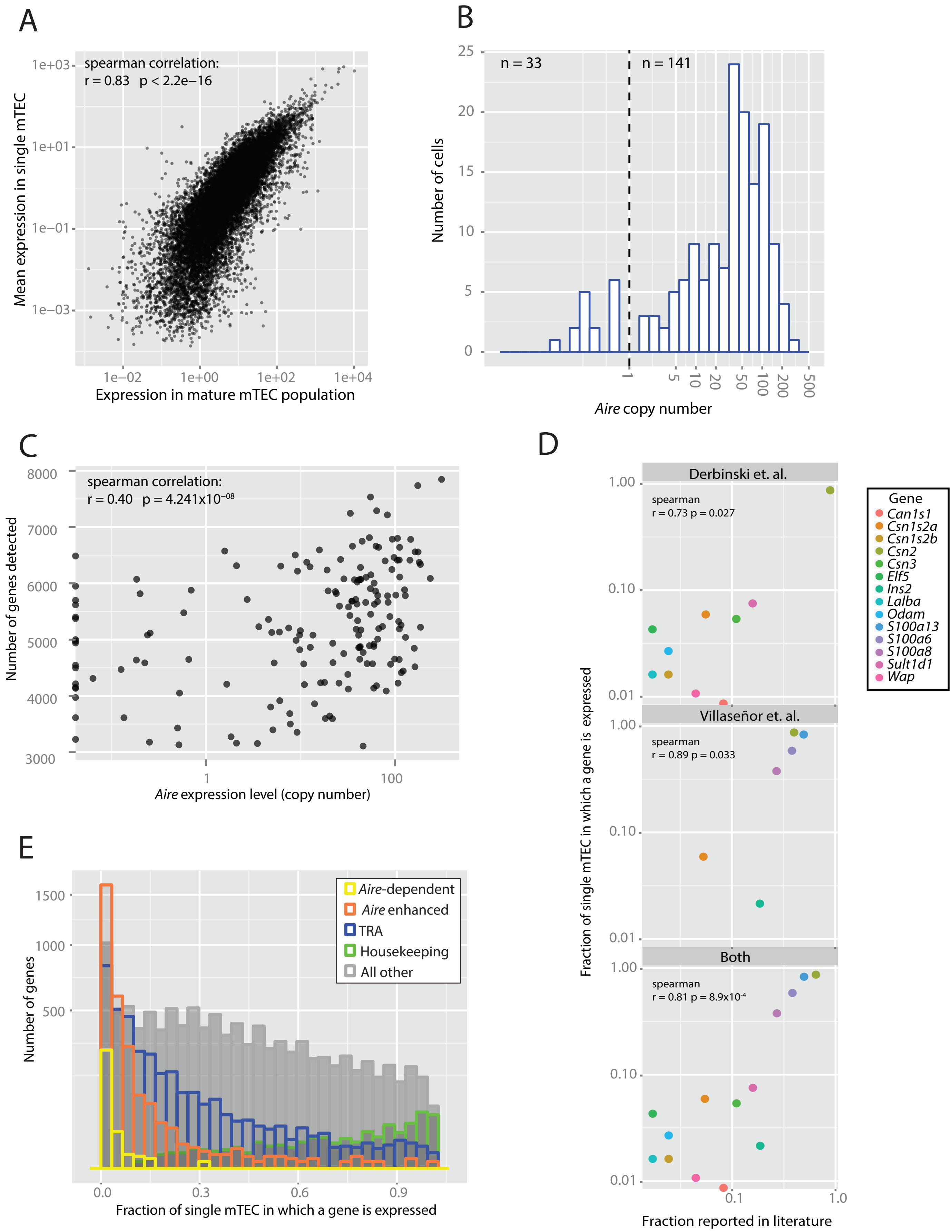

Supplementary Figure 12

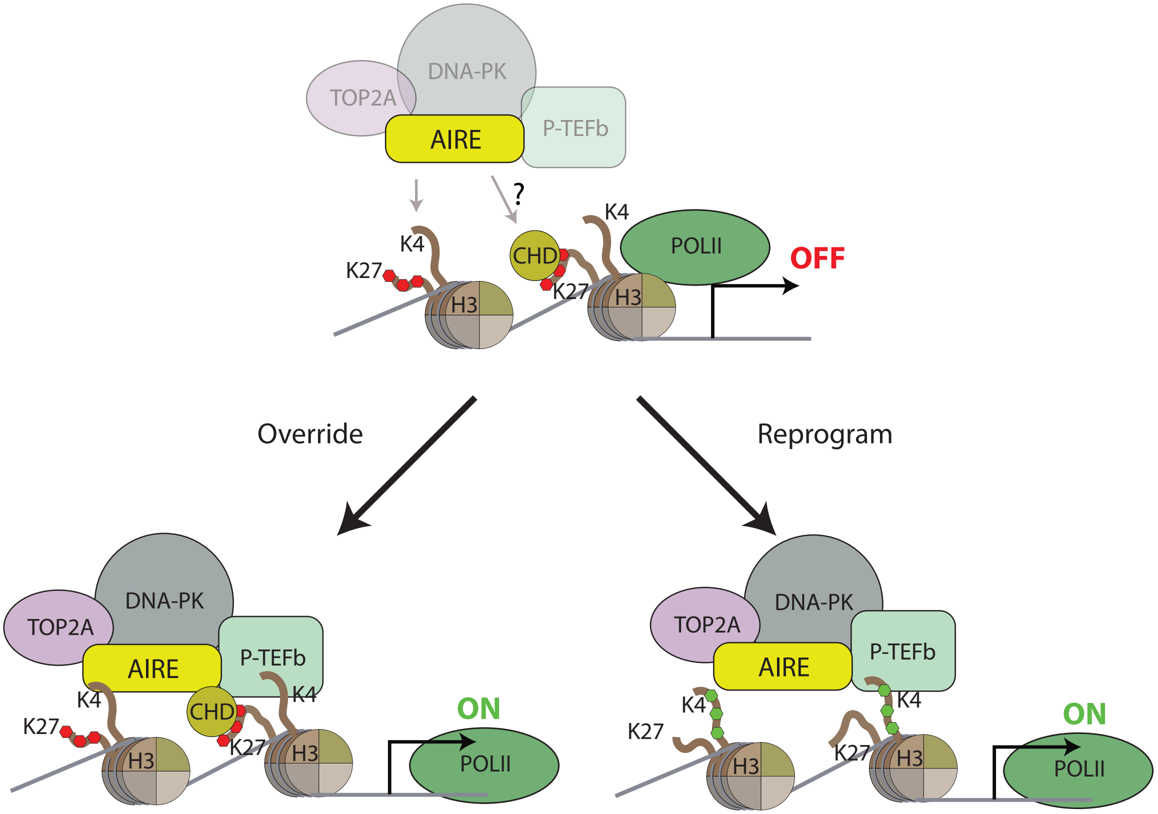

Supplementary Figure 13

Supplementary Table Legends

Supplementary Table 1: FACS sorted TEC populations analysed by RNA-seq. Two biological replicates of each of seven different populations of cortical (c) and medullary (m) TEC were isolated by fluorescence-activated cell sorting (FACS) for the given cellular phenotypes from mouse thymi. The expression of *Aire* in Fragments per Kilobase of transcript per Million fragments mapped (FPKM) was determined using the Cuffdiff algorithm(Trapnell et al. 2010). *Aire* expression is not shown for the homozygous knockout mTEC populations as this is almost entirely limited to the unmodified 5'UTR (Supplementary Fig. 2B).

Supplementary Table 2: Expression of genes in TEC and dynamic step tissue specificity calls. The table shows calculated and Cuffdiff FPKMs for the seven TEC populations (Supplementary Methods, Supplementary Table 1). Expression specificity was determined using the GNF GeneAtlas and the dynamic step method (Supplementary Methods and Supplementary Fig. 6). NRE: Non-restricted expression, TRE: tissue restricted expression (1-5 tissue groups, GNF GeneAtlas), TRE+NRE: Conflicting evidence for tissue specificity from the GNF GeneAtlas. The expression levels reported for *Aire* in the homozygous mutant mTEC population should be interpreted with caution as they largely arise from the unmodified 5'UTR. Local FDR scores associated with the calculated FPKMs are provided in a separate worksheet (Methods, Supplementary Fig. 3).

Supplementary Table 3: Genes differentially expressed between all pair-wise combinations of the seven sequenced TEC populations: Genes differentially expressed at greater than 2 fold are shown (5%FDR, DESeq analysis, see Supplementary Methods, Supplementary Fig. 4A &B). FDR: False Discovery Rate. BH: Benjamini-Hochberg.

Supplementary Table 4: KEGG pathways enriched in genes differentially expressed between three fundamental TEC types (cTEC, immature mTEC and mature mTEC). Here, *Aire* negative mature mTEC are used to represent mature mTEC in order to avoid the confounding effects of *Aire* expression in these cells (which are otherwise very similar, Supplementary Fig. 8D). For each KEGG pathway, the number of pathway genes significantly differentially expressed in each pairwise comparison is shown (No. obs.) along with the enrichment of the pathway among the differentially expressed genes (Fold) and the (per pairwise comparison) Benjamini-Hochberg (BH) false discovery rate (FDR). The KEGG ID and KEGG pathway columns contain hyperlinks to worksheets showing the expression of pathway members (that are significantly differentially expressed in at least one comparison) in these TEC types. FPKM values (Cuffdiff) in individual worksheets are coloured to highlight low (blue) and high (red) expression values using arbitrary scales.

Supplementary Table 5: Genes whose expression is characteristic of three fundamental TEC types (cTEC, immature mTEC and mature mTEC). Here, *Aire* negative mature mTEC are used to represent mature mTEC in order to avoid the confounding effects of *Aire* expression in these cells (which are otherwise very similar, Supplementary Fig. 8D). Genes characteristic of each type were identified as those expressed at an FPKM of at least 10 (Cuffdiff) and at least two fold higher (at 5%FDR, DESeq analysis, Supplementary Tables 2 & 3) than in the other two TEC types. Genes characteristic for each of the three TEC types are shown in separate worksheets as indicated.

Supplementary Table 1: FACS sorted TEC populations analysed by RNA-seq.

	TEC population	Age (weeks)	*Aire* locus	Cellular phenotype	*Aire* FPKM
1	cTEC	1	wildtype	$CD45^-$ $EpCAM^+$ $MHCII^+$ $Ly51^+$ UEA^-	1.44
2	mTEC	1	wildtype	$CD45^-$ $EpCAM^+$ $MHCII^+$ $Ly51^-$ UEA^+	405.6
3	immature mTEC	4	wildtype	$CD45^-$ $EpCAM^+$ $MHCII^{lo}$ $Ly51^-$ UEA^+	24.2
4	mature mTEC	4	wildtype	$CD45^-$ $EpCAM^+$ $MHCII^{hi}$ $Ly51^-$ UEA^+	546.0
5	mature *Aire*-negative mTEC	4	GFP/GFP (GFP -ve)	$CD45^-$ $EpCAM^+$ $MHCII^{hi}$ $Ly51^-$ UEA^+ GFP^-	-
6	mature *Aire*-positive mTEC	4	wildtype/GFP (GFP +ve)	$CD45^-$ $EpCAM^+$ $MHCII^{hi}$ $Ly51^-$ UEA^+ GFP^+	406.1
7	mature *Aire*-knockout (*Aire*-KO) mTEC	4	GFP/GFP (GFP +ve)	$CD45^-$ $EpCAM^+$ $MHCII^{hi}$ $Ly51^-$ UEA^+ GFP^+	-

Supplementary Tables 2-5 are available separately as excel files.

Supplemental References

Abramson J, Giraud M, Benoist C, Mathis D. 2010. Aire's partners in the molecular control of immunological tolerance. *Cell* **140**(1): 123-135.

Anders S, Huber W. 2010. Differential expression analysis for sequence count data. *Genome Biol* **11**(10): R106.

Derbinski J, Pinto S, Rösch S, Hexel K, Kyewski B. 2008. Promiscuous gene expression patterns in single medullary thymic epithelial cells argue for a stochastic mechanism. *Proc Natl Acad Sci U S A* **105**(2): 657-662.

Efron B. 2005. Local False Discovery Rates.

Gaetani M, Matafora V, Saare M, Spiliotopoulos D, Mollica L, Quilici G, Chignola F, Mannella V, Zucchelli C, Peterson P et al. 2012. AIRE-PHD fingers are structural hubs to maintain the integrity of chromatin-associated interactome. *Nucleic Acids Res* **40**(22): 11756-11768.

Gardner JM, Devoss JJ, Friedman RS, Wong DJ, Tan YX, Zhou X, Johannes KP, Su MA, Chang HY, Krummel MF et al. 2008. Deletional tolerance mediated by extrathymic Aire-expressing cells. *Science* **321**(5890): 843-847.

Käll L, Storey JD, Noble WS. 2009. QVALITY: non-parametric estimation of q-values and posterior error probabilities. *Bioinformatics* **25**(7): 964-966.

Koh AS, Kuo AJ, Park SY, Cheung P, Abramson J, Bua D, Carney D, Shoelson SE, Gozani O, Kingston RE et al. 2008. Aire employs a histone-binding module to mediate immunological tolerance, linking chromatin regulation with organ-specific autoimmunity. *Proc Natl Acad Sci U S A* **105**(41): 15878-15883.

Martin D, Brun C, Remy E, Mouren P, Thieffry D, Jacq B. 2004. GOToolBox: functional analysis of gene datasets based on Gene Ontology. *Genome Biol* **5**(12): R101.

Org T, Chignola F, Hetényi C, Gaetani M, Rebane A, Liiv I, Maran U, Mollica L, Bottomley MJ, Musco G et al. 2008. The autoimmune regulator PHD finger binds to non-methylated histone H3K4 to activate gene expression. *EMBO Rep* **9**(4): 370-376.

Oven I, Brdicková N, Kohoutek J, Vaupotic T, Narat M, Peterlin BM. 2007. AIRE recruits P-TEFb for transcriptional elongation of target genes in medullary thymic epithelial cells. *Mol Cell Biol* **27**(24): 8815-8823.

Ramsköld D, Wang ET, Burge CB, Sandberg R. 2009. An abundance of ubiquitously expressed genes revealed by tissue transcriptome sequence data. *PLoS Comput Biol* **5**(12): e1000598.

Sims RJ, Belotserkovskaya R, Reinberg D. 2004. Elongation by RNA polymerase II: the short and long of it. *Genes Dev* **18**(20): 2437-2468.

Suzuki R, Shimodaira H. 2006. Pvclust: an R package for assessing the uncertainty in hierarchical clustering. *Bioinformatics* **22**(12): 1540-1542.

Trapnell C, Williams BA, Pertea G, Mortazavi A, Kwan G, van Baren MJ, Salzberg SL, Wold BJ, Pachter L. 2010. Transcript assembly and quantification by RNA-Seq reveals unannotated transcripts and isoform switching during cell differentiation. *Nat Biotechnol* **28**(5): 511-515.

Villaseñor J, Besse W, Benoist C, Mathis D. 2008. Ectopic expression of peripheral-tissue antigens in the thymic epithelium: probabilistic, monoallelic, misinitiated. *Proc Natl Acad Sci U S A* **105**(41): 15854-15859.

Yang S, Bansal K, Lopes J, Benoist C, Mathis D. 2013. Aire's plant homeodomain(PHD)-2 is critical for induction of immunological tolerance. *Proc Natl Acad Sci U S A* **110**(5): 1833-1838.
